# Tree Cover and Temperature Shape the Distribution of Epiphytic *Pleurozia* in Asia: Forest Havens in a Warming Climate

**DOI:** 10.1002/ece3.73657

**Published:** 2026-06-01

**Authors:** Liangtao Huang, Feihan Chen, Yuwei Su, Zhongyi Sun, Xiao Huang, Huimin Lin, De Gao, Lina Zhang

**Affiliations:** ^1^ Ministry of Education Key Laboratory for Genetics and Germplasm Innovation of Tropical Special Forest Trees and Ornamental Plant Hainan University Haikou China; ^2^ School of Ecology, Hainan University Haikou China; ^3^ Key Laboratory of Tropical Island Land Surface Processes and Environmental Changes of Hainan Province, School of Geography and Environmental Sciences Hainan Normal University Haikou China; ^4^ International Joint Center for Terrestrial Biodiversity Around South China Sea of Hainan Province Hainan University Haikou China

**Keywords:** climate warming, cloud forests, epiphytic bryophytes, MaxEnt, species distribution modeling

## Abstract

*Pleurozia* (Pleuroziaceae, Marchantiophyta) is an evolutionarily and ecologically significant genus of epiphytic liverworts, primarily distributed in tropical and subtropical montane forests. However, the effects of climate change on its distribution remain poorly understood. In this study, we employed the MaxEnt model to predict the potential distribution patterns of *Pleurozia* in Asia at both the genus and species levels under current and future climate scenarios (SSP1‐2.6 and SSP5‐8.5 for 2050 and 2070). The results revealed that evergreen broadleaf forest cover, temperature variability (annual and diurnal ranges), and altitude were identified as the dominant environmental drivers, indicating that the distribution of *Pleurozia* is jointly governed by macroclimate and forest‐mediated microclimatic buffering. Under current conditions, suitable habitats for *Pleurozia* are mainly concentrated in tropical and subtropical Asia, with highly suitable habitats closely associated with montane cloud forests, which provide stable and humid microclimates essential for epiphytic bryophytes. Future projections indicate a slight expansion of the total suitable habitat under all scenarios, with the largest extent under SSP1‐2.6 and range margins generally extending toward higher latitudes. However, centroid displacement at the genus level remains relatively limited, suggesting that *Pleurozia* may persist in tropical regions by relying on stable forest habitats and microrefugia. At the species level, all centroids shift northward, but responses differ in spatial pattern, habitat area, and migration distance, indicating divergent distribution dynamics under future climate scenarios. These findings suggest that *Pleurozia* may retain some resilience under mid‐ to late‐century climate change, but the persistence of its suitable habitats will depend strongly on the stability and continuity of evergreen broadleaf and montane cloud forests as vital “forest havens”. This study provides a theoretical basis for understanding the ecological responses of epiphytic bryophytes to climate change and offers valuable insights for biodiversity conservation and sustainable management of tropical and subtropical forest ecosystems.

## Graphical abstract

This study uses an optimized MaxEnt model to predict the potential distribution of *Pleurozia* across Asia under current and future climate scenarios. Our results show that evergreen broadleaf forest cover, temperature annual range, mean diurnal range, and altitude are the key environmental determinants, and that suitable habitats are projected to expand slightly with a northward shift under future warming. These findings highlight the potential short‐term adaptability of *Pleurozia* and underscore the importance of conserving evergreen broadleaf forests, especially cloud forests, as ecological refugia.

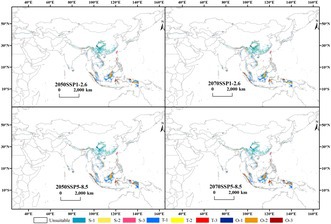

## 1 Introduction

Climate change is a major driver of global biodiversity change, affecting ecosystem processes and reshaping species distributions across spatial scales (Bellard et al. 2012; Müller et al. 2017). At large spatial scales, species distributions are primarily shaped by abiotic factors such as climate, topography, and soil, with climate generally considered the most critical (Zhu et al. 2013; Cao et al. 2019). Consequently, understanding the effects of climate change on species distributions has become a central focus in studies of global biodiversity patterns (Jump and Peñuelas 2005). These issues are particularly critical in tropical and subtropical regions, which contain many of the world's biodiversity hotspots, characterized by exceptionally high endemism and concurrent severe habitat loss (Myers et al. 2000).

The tropics harbor the highest terrestrial biodiversity (Dowle et al. 2013). However, without effective mitigation measures, tropical surface temperatures are projected to rise by up to 3°C by the end of the century (Müller et al. 2017), posing significant risks to tropical forest ecosystems (Clark et al. 2003; Feeley et al. 2007). Evidence from previous studies suggests that tropical trees may already be nearing their upper thermal limits for photosynthesis; exceeding these thresholds could substantially reduce CO_2_ assimilation and inhibit growth (Doughty and Goulden 2008). In tropical and subtropical Asia, evergreen broadleaf forests dominate regional vegetation and play a fundamental role in water regulation, carbon storage, and biodiversity maintenance (Ashton and Zhu 2020; Zhu 2021). These forests also regulate local climate through carbon sequestration and evaporative cooling (Bonan 2008), creating stable, humid understory microhabitats that are particularly favorable for epiphytic communities, including bryophytes (Sporn et al. 2010).

Epiphytic bryophytes, growing on living shrubs, tree trunks, branches, or leaves, constitute an important component of tropical and subtropical forest ecosystems, particularly abundant in tropical rainforests and montane cloud forests (Smith 1982). Approximately 21,000 bryophyte species have been identified worldwide (Zhu et al. 2022), with more than 6000 epiphytic species recorded in tropical regions, accounting for nearly 60% of regional bryophyte diversity in these areas (Zotz and Bader 2009; Norhazrina et al. 2017). These unique plants play a crucial role in forest ecosystems. By capturing water from precipitation and cloud moisture, they help maintain canopy humidity during dry periods (Pypker et al. 2006). They also contribute to nutrient and material cycling through processes such as precipitation interception, atmospheric deposition, and symbiotic associations with nitrogen‐fixing organisms (Lindo and Whiteley 2011). In addition, epiphytic bryophytes enhance forest structural complexity and provide essential habitats for a variety of plants and animals, thus promoting biodiversity (Sporn et al. 2010).

Bryophytes are characterized by their small size, lack of true roots and vascular tissues, often single cell‐layered leaves, and absence of a protective cuticle. These traits make them primarily dependent on atmospheric inputs, such as rain, dew, and airborne particulates, for water and nutrient acquisition (Pott and Turpin 1996). As a result, they are highly sensitive to environmental fluctuations, with physiological responses to temperature and moisture often more pronounced than those of vascular plants (Vanderpoorten and Goffinet 2009), and are therefore widely recognized as effective indicators of climate change impacts on biodiversity (Wu et al. 2002). This sensitivity is particularly evident in epiphytic bryophytes, which are typically associated with cool, humid, high‐precipitation environments and are further constrained by host tree characteristics and forest continuity (Zhao et al. 2020; Xu et al. 2023). Their strong dependence on both microclimatic conditions and suitable substrates renders them particularly vulnerable to environmental change and highly reliant on stable forest habitats under climate change (He et al. 2016).

The genus *Pleurozia*, classified under the Phylum Marchantiopsida, class Jungermanniopsida, order Pleuroziales, and family Pleuroziaceae, comprises 12 recognized species according to the latest world checklist of liverworts (Söderström et al. 2016). Species of *Pleurozia* are primarily epiphytic, typically growing on moist, shaded tree trunks in high‐altitude montane rainforests of tropical and subtropical regions, usually at elevations above 2000 m (Thiers 1993). Notably, *Pleurozia* is characterized by a two‐sided apical cell, in contrast to the typical three‐sided apical cell found in most leafy liverworts (Crandall‐Stotler 1976; Thiers 1993; Müller 2013). This distinctive feature positions *Pleurozia* as a unique lineage at the evolutionary transition between simple thalloid and leafy liverworts (He‐Nygrén et al. 2006), highlighting its significant value in liverwort phylogenetic studies. Meanwhile, as epiphytes, *Pleurozia* species and their montane cloud forest habitats are both highly vulnerable to climate changes. Consequently, they have the potential to serve as indicators for monitoring fluctuations in environmental conditions and forest ecosystems, while also facing an increased risk of becoming endangered. Their conservation has received growing global attention: for instance, *P. purpurea* and *P. subinflata* are listed as Near Threatened (NT) in the Red List of China's Biodiversity—Higher Plants Volume (2020) (Ministry of Ecology and Environment and Chinese Academy of Sciences 2023) and *P. paradoxa* is categorized as vulnerable in the Red Book of Bryophytes of Colombia (Aroca‐Gonzalez et al. 2021).

Current research on the genus *Pleurozia* primarily focuses on taxonomy, morphology, and phylogeny; however, limited attention has been given to its potential distribution and response to climate change. While similar modeling approaches have been increasingly used for other bryophyte groups, comparable distributional assessments remain scarce for epiphytic liverwort genera such as *Pleurozia* (Abay and Gül 2024, 2025a, 2025b). Focusing on the genus *Pleurozia* across Asia and on the four individual species with sufficiently predictable distributions, this study aims to address the following objectives: (1) to predict the distribution patterns and classify suitability zones for potential *Pleurozia* habitats in Asia at both the genus and species levels under current climatic conditions; (2) to identify the key environmental factors influencing habitat suitability; (3) to project future distributional shifts and center‐of‐mass migration trajectories under various climate scenarios. The findings of this study will offer valuable insights into the potential habitats of *Pleurozia* and the main environmental drivers shaping the distribution of epiphytic bryophytes. Furthermore, the results may contribute to conservation strategies and sustainable management of epiphytic bryophyte diversity in tropical and subtropical rainforests.

## 2 Materials and Methods

### 2.1 Species Distribution Data

According to the World Checklist of Hornworts and Liverworts (Söderström et al. 2016; Sukkharak 2017), the genus *Pleurozia* comprises 12 species worldwide, seven of which occur in Asia. Given that Asia is a major center of diversity for the genus and has relatively comprehensive occurrence records, it was selected as the primary focus for distribution modeling. Potential distributions were modeled at both the genus and species levels to evaluate patterns across scales. Because MaxEnt modeling generally requires at least five occurrence records to achieve reliable predictive performance, four species ( *Pleurozia acinosa* , *P. gigantea* , *P. purpurea* , and *P. subinflata* ) met this criterion and were included in the species‐level analyses.

**FIGURE 1:**
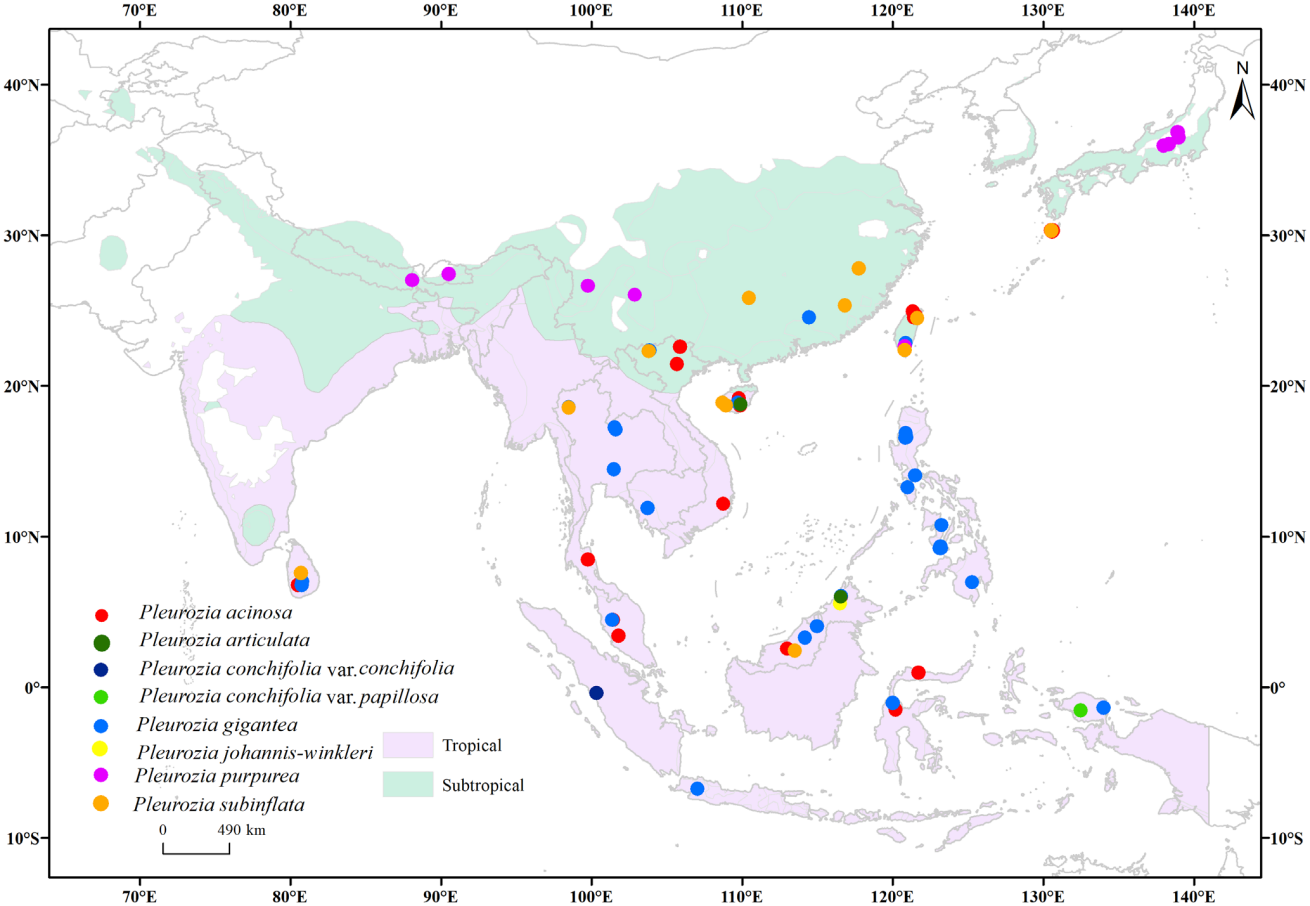
Selected valid occurrence records of *Pleurozia* species in Asia.

The species distribution data were gathered from multiple sources, including “Flora Bryophytorum Sinicorum” (Gao and Wu 2003), “Bryophyte Flora of Guangdong” (Wu and Zhang 2013), field surveys carried out by our research team in Hainan, relevant literature (Mues et al. 1991; Wang et al. 2006; Shu et al. 2016; Söderström et al. 2016; Kamada et al. 2020; Bakalin et al. 2023; Hsu et al. 2023; Sass‐Gyarmati et al. 2023), and online databases such as the Global Biodiversity Information Facility (GBIF), the Consortium of North American Bryophyte Herbaria (CNABH), and Tropicos. For records with only locality names, specific latitude and longitude coordinates were obtained using Google Earth. In total, 135 occurrence records were collected across the Asia region. To reduce sampling bias caused by the concentration of numerous occurrence records within a limited area, which can negatively affect model performance, spatially clustered or duplicate records were systematically removed. Using the ENMTools software package (Warren et al. 2021), a single occurrence record was randomly retained per grid cell, resulting in 81 valid data points for subsequent ecological niche modeling analyses (Figure 1; Table S1).

According to the Köppen–Geiger climate classification, Asia can be categorized into five major climate types: Tropical, Arid, Temperate, Cold, and Polar (Peel et al. 2007). This study specifically focuses on the Tropical and Temperate climate types, with the latter encompassing both subtropical and temperate maritime climates, as the majority of *Pleurozia* occurrences fall within these zones (Figure 1). Furthermore, within the Temperate classification, the distribution of *Pleurozia* predominantly overlaps with subtropical regions, approximately between 23.5° N and 40° N latitude. Therefore, the term “subtropical” is employed alongside “tropical” to more accurately delineate the primary suitable habitat areas for *Pleurozia*. The base map data used in this study were sourced from the world map published by the Standard Map Service, reviewed under license number GS (2016) 1666.

### 2.2 Environmental Factors

Climatic factors are widely recognized as the most significant environmental variables influencing species distribution. In particular, 19 bioclimatic variables are commonly used as the most representative for modeling and predicting species distribution. In this study, these 19 variables, along with altitude, solar radiation, and water vapor pressure (Table S2), were acquired from the WorldClim database (http://www.worldclim.org) at a spatial resolution of 2.5 arc‐minutes. Furthermore, five global 1‐km consensus land‐cover datasets (Tuanmu and Jetz 2014) and leaf area index data (Fang et al. 2019) were incorporated, considering that *Pleurozia* typically inhabits tree surfaces or areas beneath the forest canopy. The five land‐cover types (Cons1–Cons5) represent the following vegetation categories: evergreen/deciduous needleleaf trees, evergreen broadleaf trees, deciduous broadleaf trees, mixed/other trees, and shrubs (see Table S2).

Based on a total of 28 environmental variables, we predicted the potential distribution of the genus *Pleurozia* in Asia, as well as separate models for four individual species: *P. acinosa* , *P. gigantea* , *P. purpurea* , and *P. subinflata* . Projections were conducted for across three time periods: the current, 2050 (2041–2060), and 2070 (2061–2080). The future climate projections were derived from the MPI‐ESM1‐2‐HR model under the Coupled Model Intercomparison Project Phase 6 (CMIP6). First, MPI‐ESM1‐2‐HR provides a high spatial resolution (HR, high resolution) that is appropriate for capturing regional climate variability and topographically complex environments, which are critical for our study area. Second, this model has been widely validated and commonly used in ecological niche modeling, species distribution modeling, and climate change impact studies, ensuring comparability with previous research. Third, MPI‐ESM1‐2‐HR performs well in simulating temperature, precipitation, and other key bioclimatic variables across mid‐ to high‐latitude and mountainous regions, which align well with the environmental conditions of our study system. Finally, the model's outputs are readily accessible, well‐documented, and frequently employed in global and regional climate impact assessments, supporting the reliability and reproducibility of our analyses.

Two contrasting shared socioeconomic pathways (SSPs) were selected to represent the minimum and maximum greenhouse gas emission scenarios: SSP1‐2.6, a low‐emission scenario aimed at limiting global warming to below 2°C, and SSP5‐8.5, a high‐emission scenario characterized by fossil fuel‐intensive development. For future projections, only the 19 bioclimatic variables were updated to reflect projected climate conditions, whereas the remaining environmental variables, including land cover, altitude, solar radiation, water vapor pressure, and leaf area index, were held constant.

High correlations among environmental variables can lead to model overfitting; therefore, careful selection of input variables is essential (Chen et al. 2022). Initially, all 28 environmental variables listed in Table S2 were analyzed using the MaxEnt model, which was run with 10 replicates to eliminate variables with zero contribution. A correlation analysis was then performed using the Band Collection Statistics tool in ArcGIS, and a correlation heat map was generated (Figure S1). For variable pairs with a Pearson correlation coefficient of |r| > 0.7 (Dormann et al. 2013), the variable with the higher contribution in the initial model run was retained. As a result, a final set of 10 environmental variables was selected for modeling the distribution of *Pleurozia*: Bio2, Bio7, Bio15, Cons1, Cons2, Cons3, Cons4, Cons5, Alt, and Sr.

Following the same variable selection procedure, the final sets of environmental predictors retained for modeling each *Pleurozia* species were as follows. For *Pleurozia acinosa* : Bio2, Bio11, Bio15, Cons1, Cons2, Cons3, Cons4, Cons5, and Alt. For *P. gigantea* : Bio2, Bio4, Bio15, Cons2, Cons3, Cons4, Cons5, and Alt. For *P. purpurea* : Bio2, Bio7, Bio8, Bio15, Bio18, Cons3, Cons4, Cons5, Alt, Lai, and Sr. For *P. subinflata* : Bio2, Bio7, Bio8, Bio18, Bio19, Cons1, Cons3, Cons4, Alt, and Lai.

### 2.3 Model Optimization

The use of default parameters in MaxEnt models may result in suboptimal performance, often causing underfitting or limiting the model's predictive accuracy. (Yackulic et al. 2013). The predictive performance of MaxEnt is primarily influenced by two parameters: the regularization multiplier (RM) and the feature combination (FC). Parameter tuning and selecting were performed using the kuenm package in R (Cobos et al. 2019). Feature Combinations (FCs) define nonlinear relationships between species and environmental variables, ranging from simple to highly complex interactions (Fu et al. 2024). FCs comprise five features: Linear (L), Quadratic (Q), Product (P), Threshold (T), and Hinge (H), which can be combined in various ways to achieve the optimal configuration. The selection process for the optimal models was guided by several criteria: first, candidate models that demonstrated statistical significance and had a training data omission rate of ≤ 5% were identified. From this subset, the final models were selected based on delta Akaike Information Criterion (AICc) values ≤ 2, indicating these models have strong support relative to the best‐performing model (Cobos et al. 2019).

During the parameter optimization process, a total of 40 regularization multiplier values, ranging from 0.1 to 4.0, were tested. Simultaneously, 29 different feature combinations were assessed, including both individual and multiple combinations of L, Q, P, T, H, LQ, LP, LT, LH, QP, QT, QH, PT, PH, TH, LQP, LQT, LQH, LPT, LPH, QPT, QPH, QTH, PTH, LQPT, LQPH, LQTH, LPTH, and LQPTH. In total, 1160 candidate models were generated and evaluated.

### 2.4 Model Construction and Evaluation

The species occurrence data and environmental variables for each time period were imported into MaxEnt for modeling. Seventy‐five percent of the occurrence records were designated as the training set, while the remaining 25% were assigned to the test set. The model was executed with 10 replicates using the “Cross‐validate” option. The “Create response curves” feature was enabled and the output format was set to “Logistic,” with all other settings left at their default values. Model accuracy was evaluated using the Area Under the Curve (AUC) of the Receiver Operating Characteristic (ROC) curve generated by MaxEnt. The AUC values range from 0 to 1, with higher values indicating greater predictive accuracy. Values below 0.7 suggest poor performance; those between 0.7 and 0.8 indicate moderate performance, values from 0.8 to 0.9 are considered good, and values exceeding 0.9 reflect excellent predictive ability (Wang et al. 2023).

### 2.5 Division of Suitable Areas

Following previous studies (Abolmaali et al. 2018; Zhang et al. 2019; Wu et al. 2023), the predicted habitat suitability for *Pleurozia* and its four main species ( *P. acinosa* , *P. gigantea* , *P. purpurea* , and *P. subinflata* ) was classified into four categories using ArcGIS 10.7: unsuitable (< 0.2), low suitability (0.2–0.4), moderate suitability (0.4–0.6), and high suitability (> 0.6). The area of each suitability class was then quantified using the grid calculation tool in ArcGIS.

Spatial and temporal variations in suitable habitats and distribution centers of *Pleurozia* and its four species were analyzed based on the coordinates of grid cells in the model output. Distribution centers were determined by calculating the centroids of suitable areas, and shifts in these centroids were used to reflect the geographic migration of the genus and each species over time. The SDM Toolbox in ArcGIS was employed to assess current and future trends in suitable habitat areas, as well as to evaluate the spatial displacement of distribution centroids for both the genus and the species. Changes in centroid coordinates and migration distances were further quantified using ArcGIS 10.7.

## 3 Results

### 3.1 MaxEnt Model Optimization and Evaluation

When the MaxEnt model parameters were set to their default values (regularization multiplier, RM = 1; feature combination, FC = LQPH), the resulting delta.AICc was 224.0588. In contrast, the optimized model for *Pleurozia*, with RM set to 1.3 and FC restricted to L and Q, achieved a delta.AICc of 0. This indicates that the optimized MaxEnt model, with reduced complexity and minimized overfitting, provided the best fit for the data. Therefore, RM = 1.3 and FC = LQ were chosen as the optimal parameters for genus‐level modeling (Table S3). Species‐specific optimized parameters for the four *Pleurozia* species are also presented in Table S3.

### 3.2 Evaluation of the Accuracy of Model Results

The accuracy of the optimized MaxEnt model was assessed using ROC curves. Based on 81 occurrence records of *Pleurozia* and the selected environmental variables, the mean AUC value was determined to be 0.975 ± 0.0009 (mean ± SD) across 10 iterations, as shown in Figure S2. This indicates a high level of predictive performance and model reliability. Therefore, the potentially suitable distribution areas for the genus *Pleurozia* in Asia can be effectively predicted based on its occurrence data and associated environmental variables.

The optimized MaxEnt models at the species level also demonstrated high accuracy. As shown in Figure S2, ROC curve evaluation over 10 iterations yielded AUC values of 0.977 ± 0.0022 for *P. acinosa* , 0.981 ± 0.0010 for *P. gigantea* , 0.985 ± 0.0022 for *P. purpurea* , and 0.973 ± 0.0091 for *P. subinflata* (mean ± SD). The consistently high AUC values confirm the excellent predictive performance and reliability of the species‐level models.

### 3.3 Key Environmental Factors Shaping the Distribution

#### 3.3.1 The Genus *Pleurozia*

The percent contribution (Pc) of each environmental variable was calculated using the optimized MaxEnt model with iterative algorithms. The analysis identified four key variables (each contributing more than 10.0%) as the most significant contributors to the geographic distribution pattern of *Pleurozia* in Asia (Table S4): evergreen broadleaf trees (Cons2), temperature annual range (Bio7), mean diurnal range (Bio2), and altitude (Alt). These factors collectively accounted for 91.0% of the total contribution. Furthermore, the Jackknife test (Figure S3) demonstrated that when variables were evaluated individually, Cons2, Bio7, and Bio2 yielded the highest regularized training gain values (all > 1.0).

Environmental factor response curves illustrate the relationships between habitat suitability and individual environmental variables, thereby aiding in the understanding of a species' ecological niche. Figure S4 presents the response curves of the four key environmental variables influencing *Pleurozia* distribution. Occurrence probability increases with evergreen broadleaf forest cover (Cons2) but decreases when temperature annual range (Bio7) and mean diurnal range (Bio2) exceed their optimal thresholds, indicating a preference for thermally stable habitats. Altitude shows a unimodal relationship with occurrence probability, increasing to a peak at approximately 2134 m before gradually declining at higher elevations. Based on a probability threshold of > 0.5 for favorable growth conditions (Zhong et al. 2023), the suitable ranges of these variables are summarized in Table S4.

#### 3.3.2 Four *Pleurozia* Species

Species‐specific modeling revealed a more nuanced pattern of ecological niche differentiation among the four *Pleurozia* species. The dominant environmental variables, their percent contributions (Pc), and suitable ranges are presented in Table S5, and their relative importance is further supported by Jackknife tests (Figure S5). Environmental response curves for the four species are shown in Figures S6–S9.

The results showed that evergreen broadleaf forest cover (Cons2), leaf area index (Lai), mean diurnal range (Bio2), annual temperature range (Bio7), and altitude were key environmental variables, each with contributions (Pc) > 15% in at least two species. Temperature seasonality (Bio4), representing seasonal thermal variation, was also an important variable, but only for *P. gigantea* . Mean temperature of coldest quarter (Bio11) and Precipitation of warmest quarter (Bio18) were identified only for *P. acinosa* and *P. purpurea* , respectively. Among the four species, *P. purpurea* exhibited the broadest suitable ranges of annual temperature variation (Bio7) and altitude, suggesting greater tolerance to thermal variability and a broader elevational niche. Overall, despite interspecific variation in the dominant drivers, the distributions of the four *Pleurozia* species were shaped primarily by forest cover and structure, temperature variability, and altitude.

### 3.4 Potential Habitat Areas in the Current Period

#### 3.4.1 The Genus *Pleurozia*

**FIGURE 2:**
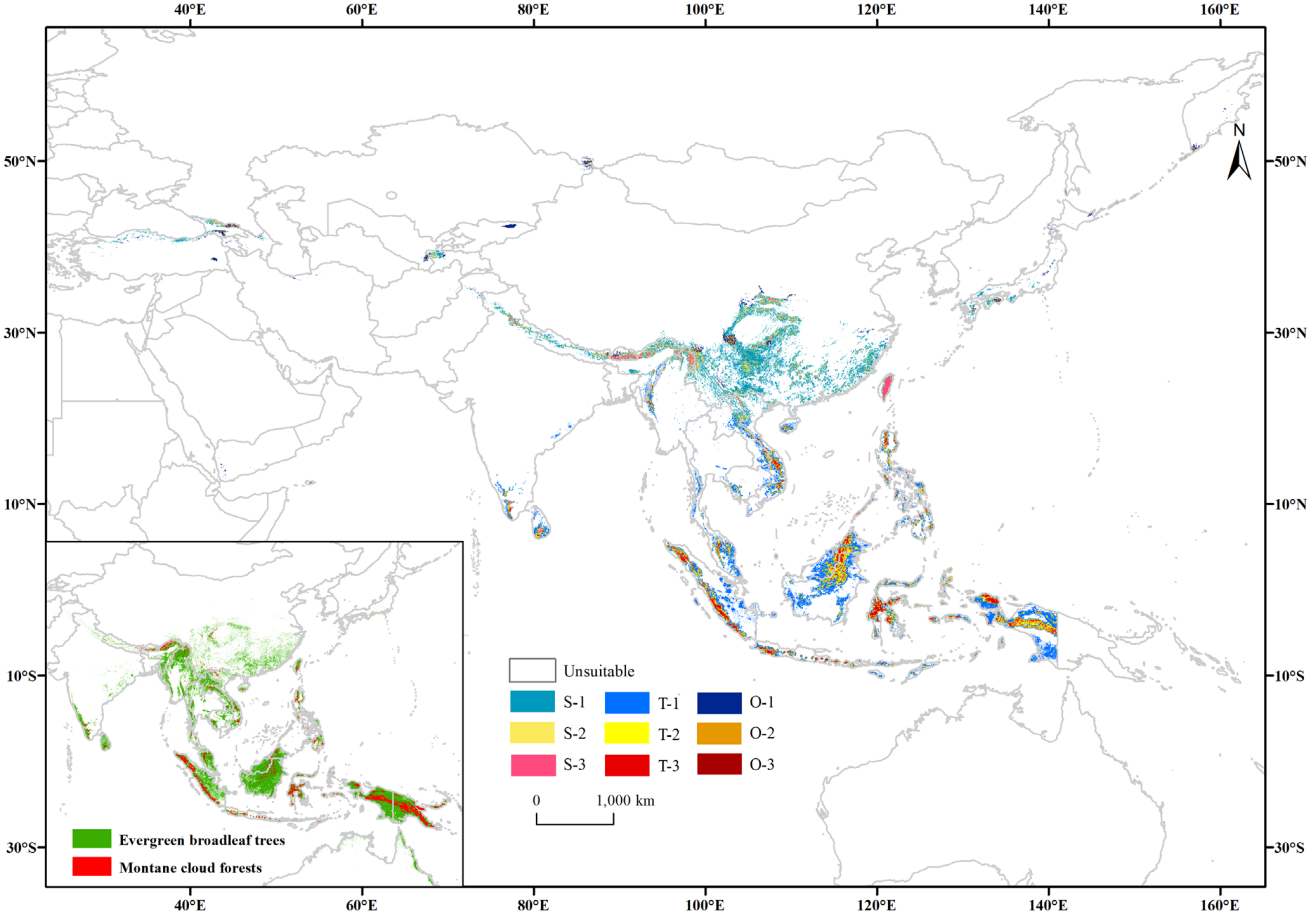
Potential habitat suitability for *Pleurozia* under current climatic conditions. S, subtropical; T, tropical; O, other regions; 1, low suitability; 2, moderate suitability; 3, high suitability (i.e., S‐1, S‐2, S‐3; T‐1, T‐2, T‐3; O‐1, O‐2, O‐3).

Based on the results of the MaxEnt model analysis, the current potential geographical distribution of *Pleurozia* is shown in Figure 2. The potential suitable areas in the current period are primarily concentrated in the following regions: the islands of Southeast Asia; southern to southwestern China and Japan in East Asia; and the Himalayan region, southern India, and Sri Lanka in South Asia. Additionally, a smaller number of suitable areas extend to the northern mountainous regions of Türkiye and the Caucasus in West Asia, with a few predicted areas scattered in Central and Northern Asia. The potential distribution areas largely overlap with those of evergreen broadleaf forests, a significant environmental factor contributing to the distribution of *Pleurozia* (Cons2 in Section 3.3; see Figure 2), which aligns well with *Pleurozia*'s preference for epiphytic habitats on trees. Furthermore, this potential distribution closely matches the species' actual geographical range, further indicating that the model developed in this study effectively simulates the suitable habitats for *Pleurozia*.

The total suitable area of *Pleurozia* in Asia was approximately 187.89 × 10^4^ km^2^, accounting for 4.21% of the continent's terrestrial area (Table S6). Low‐, moderate‐, and high‐suitability habitats accounted for 65.58%, 23.71%, and 10.72% of the total suitable area, respectively. Tropical and subtropical regions comprised 55.94% and 39.56% of the total suitable area, respectively, with tropical regions supporting a greater extent of low‐, moderate‐, and high‐suitability habitats overall. These results indicate that *Pleurozia* suitable habitats in Asia are predominantly concentrated in tropical regions. High‐suitability areas are mainly distributed in the Malay Archipelago, Vietnam, Myanmar, the Himalayas, Hainan, Taiwan, and southwestern China, with additional scattered patches in southern India, southern Sri Lanka, the Caucasus, and southern Japan. Most of these highly suitable areas coincide with tropical or subtropical montane cloud forests (refer to Figure 2; Karger et al. 2021).

#### 3.4.2 Four *Pleurozia* Species

**FIGURE 3:**
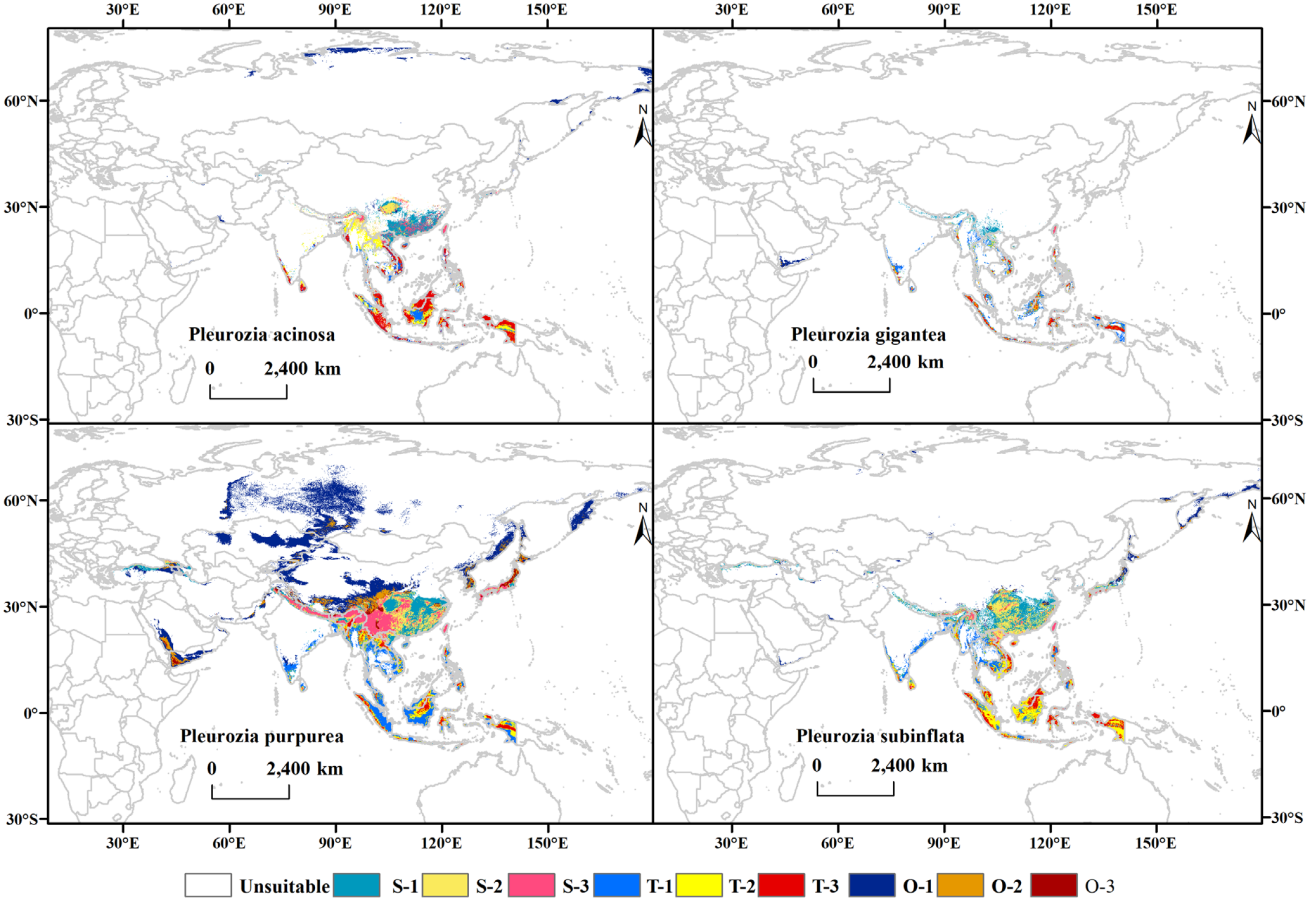
Potential habitat suitability for four *Pleurozia* species under current climatic conditions. S, subtropical; T, tropical; O, other regions; 1, low suitability; 2, moderate suitability; 3, high suitability (i.e., S‐1, S‐2, S‐3; T‐1, T‐2, T‐3; O‐1, O‐2, O‐3).

Based on the predictions of the MaxEnt model, the current potential suitable distribution patterns of the four *Pleurozia* species in Asia are shown in Figure 3, and the areas of suitable habitats for each species are presented in Table S7. In terms of spatial patterns, the moderately and highly suitable habitats of the four species are largely concentrated and overlapping across tropical and subtropical Asia, mainly in the mountainous regions of southwestern and southern China, Southeast Asia, the eastern Himalayas, and the coastal mountains of India. Among the four species, *P. gigantea* has the smallest suitable area (148.09 × 10^4^ km^2^), with moderately and highly suitable habitats primarily occurring in the tropics. By contrast, *P. acinosa* , *P. subinflata* , and *P. purpurea* show broader potential distributions, extending into temperate regions. Notably, *P. purpurea* has the largest suitable area (1083.09 × 10^4^ km^2^), with its northward extension into Russia consisting predominantly of low‐suitability habitats.

From the perspective of climatic zone distribution (based on the combined subtropical S‐series and tropical T‐series suitability categories shown in Figure 3 and Table S7), *P. gigantea* is predominantly tropical, with 78.93% of its suitable area occurring in the tropics. *P. acinosa* and *P. subinflata* are also mainly associated with tropical regions, where tropical suitable areas account for 54.29% and 47.63% of their total suitable areas, respectively, compared with 31.30% and 39.64% in subtropical regions. In contrast, *P. purpurea* shows a stronger association with subtropical regions, with subtropical suitable areas (26.55%) slightly exceeding tropical areas (24.11%). When considering only highly suitable habitats, tropical dominance remains evident for *P. gigantea* (tropical vs. subtropical: 16.92% vs. 1.09%), *P. acinosa* (29.26% vs. 6.94%), and *P. subinflata* (13.13% vs. 3.89%), whereas *P. purpurea* exhibits a stronger subtropical tendency (3.30% vs. 9.45%).

### 3.5 Potential Habitat Areas Under Future Climate Scenarios

#### 3.5.1 The Genus *Pleurozia*

**FIGURE 4:**
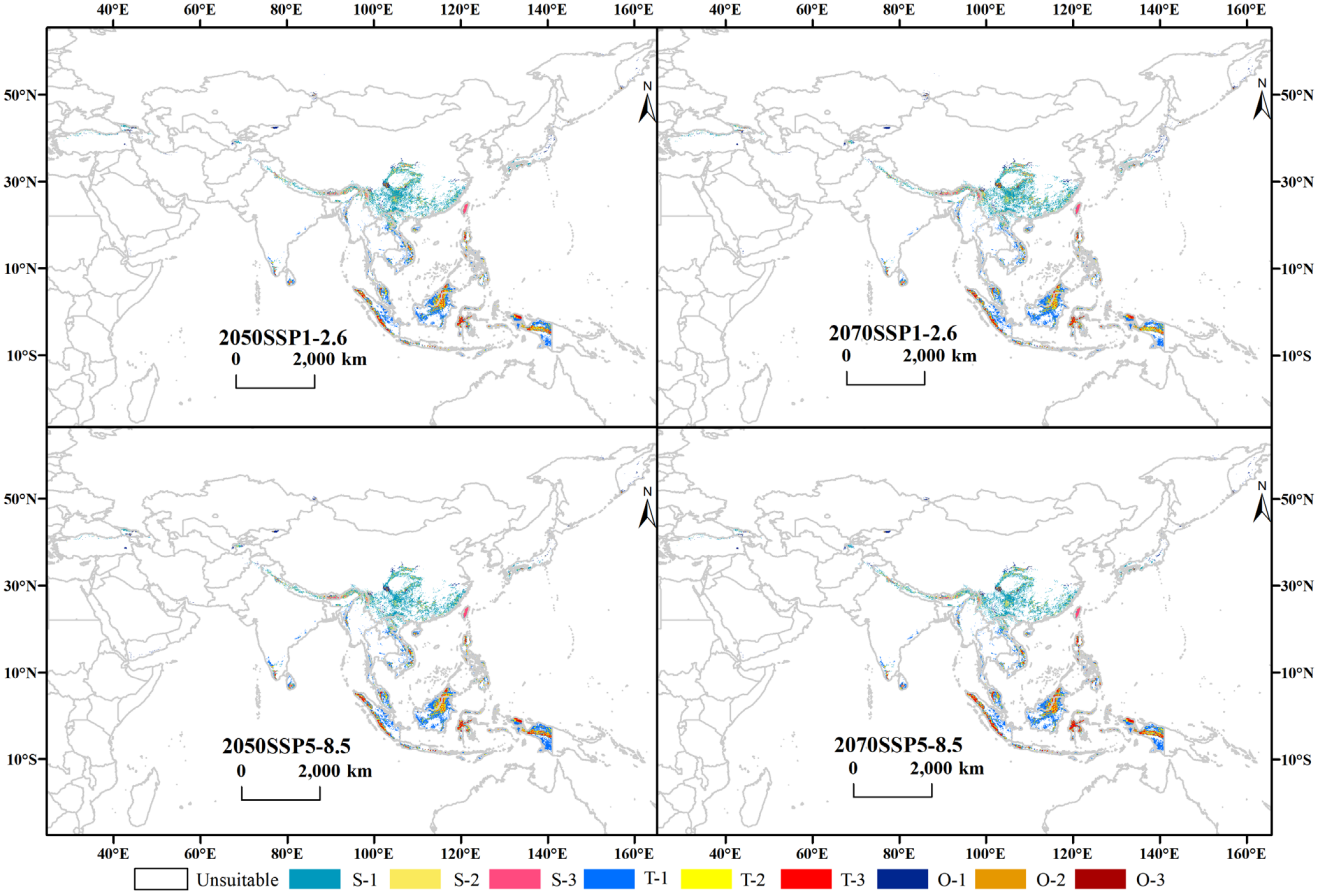
Potential habitat suitability for *Pleurozia* under future climate scenarios. S, subtropical; T, tropical; O, other regions; 1, low suitability; 2, moderate suitability; 3, high suitability (i.e., S‐1, S‐2, S‐3; T‐1, T‐2, T‐3; O‐1, O‐2, O‐3).

**FIGURE 5:**
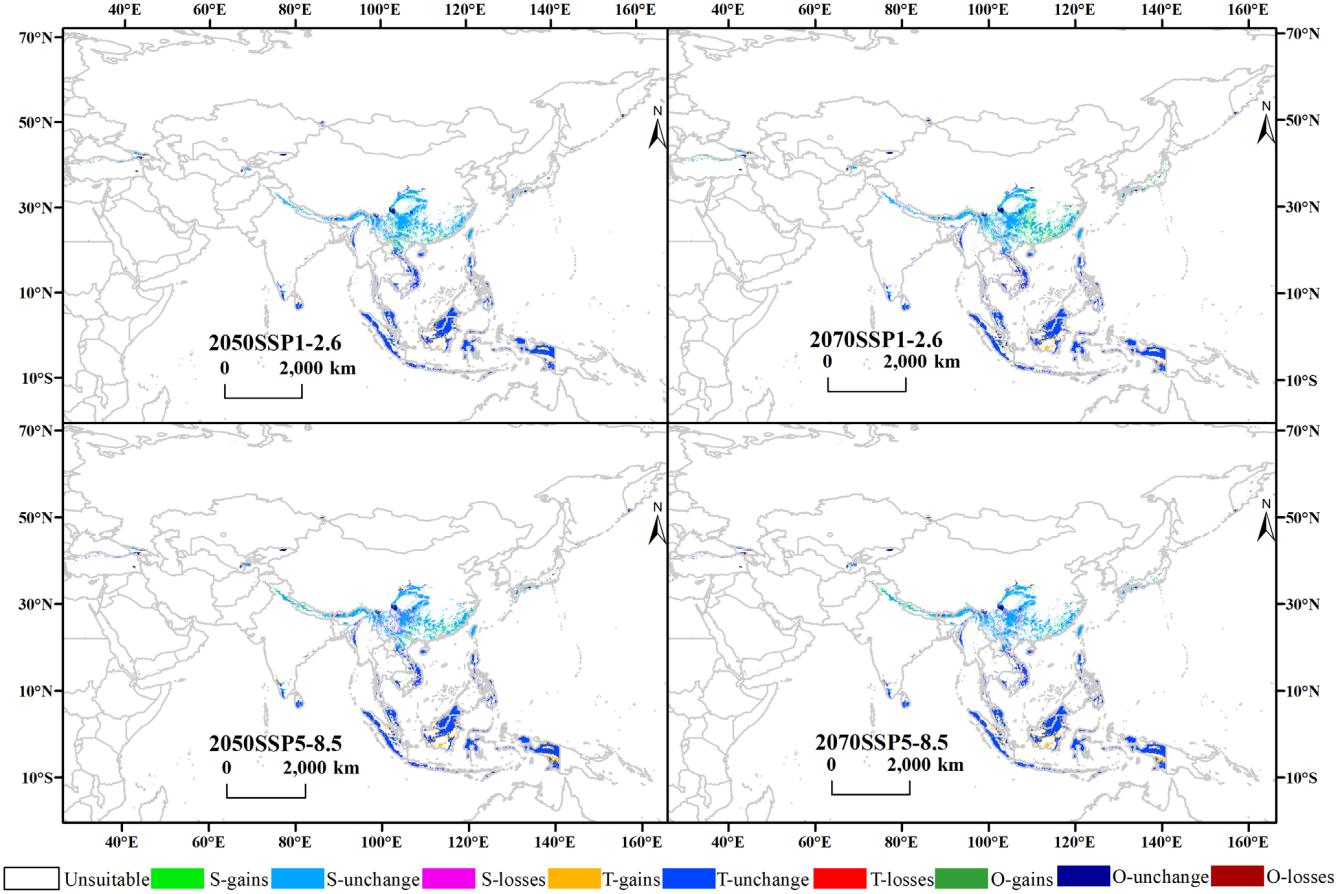
Changes in suitable habitats of *Pleurozia* over time relative to the current climate model. S, subtropical; T, tropical; O, other regions; gains, newly suitable areas; unchang, areas with no change in suitability; losses, areas that become unsuitable.

Under the two future climate change scenarios, when considering only climate‐related factors, the overall distribution of suitable habitats for *Pleurozia* remains relatively stable (Figure 4), with a slight tendency to expand toward higher latitudes in Asia (Figure 5).

It illustrates that, as time progresses and climate scenarios change, the potential suitable zone stretches from Vietnam northward into southern China and spreads westward along the Himalayas. Particularly under the 2070 SSP1‐2.6 scenario, suitable habitats are projected to expand northeastward to Honshu Island in Japan and northwestward to the northern mountains of Türkiye. The loss of suitable habitats varies among climate scenarios, with more pronounced losses under the SSP5‐8.5 scenario occurring from southwestern China to the Indochina Peninsula (mainland Southeast Asia) and in the northern mountainous regions of Türkiye. In contrast, suitable habitats in equatorial Southeast Asian islands show varying degrees of expansion (see Figure 5).

In terms of suitable habitat areas, the analysis reveals a consistent, slightly increasing trend in the total suitable habitat area, as well as in the distribution of low‐, moderate‐, and high‐suitability habitats for *Pleurozia* across all future time periods and climate scenarios, compared with the current period (Table S6). But the increases under different scenarios are primarily driven by the expansion of low‐suitability habitats, with comparatively smaller increases in moderate‐ and high‐suitability habitats. The most favorable climate scenario for *Pleurozia* seems to be SSP1‐2.6, under which the total suitable habitat area is projected to reach 214.44 × 10^4^ km^2^ by the 2070s, representing an increase of 14.13% relative to the current period.

In addition, subtropical suitable habitats exhibit a greater increase under the SSP1‐2.6 scenario than under SSP5‐8.5. The total area of subtropical suitable habitats, along with the areas of low‐, moderate‐, and high‐suitability classes, reaches its maximum in the 2070s (Table S6). In contrast, tropical suitable habitats are more extensive under SSP5‐8.5, whereas the area of highly suitable subtropical habitats under SSP5‐8.5 declines relative to the present, with a reduction of 15.21% in 2050 and 11.27% in 2070.

#### 3.5.2 Four *Pleurozia* Species

**FIGURE 6:**
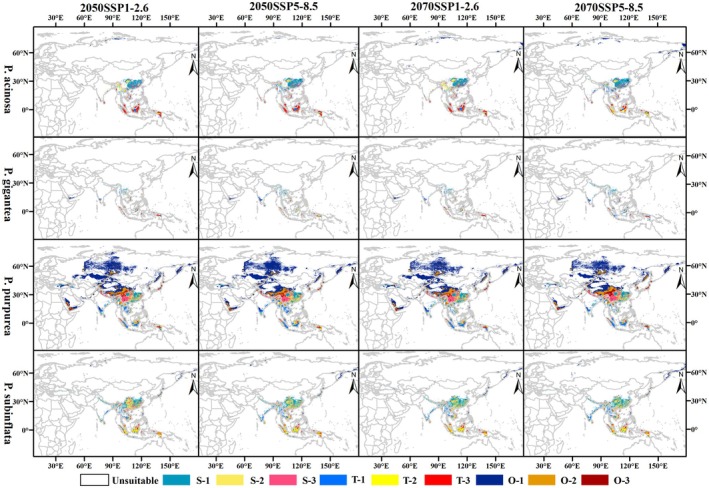
Potential habitat suitability for four *Pleurozia* species under future climate scenarios. S, subtropical; T, tropical; O, other regions; 1, low suitability; 2, moderate suitability; 3, high suitability (i.e., S‐1, S‐2, S‐3; T‐1, T‐2, T‐3; O‐1, O‐2, O‐3).

**FIGURE 7:**
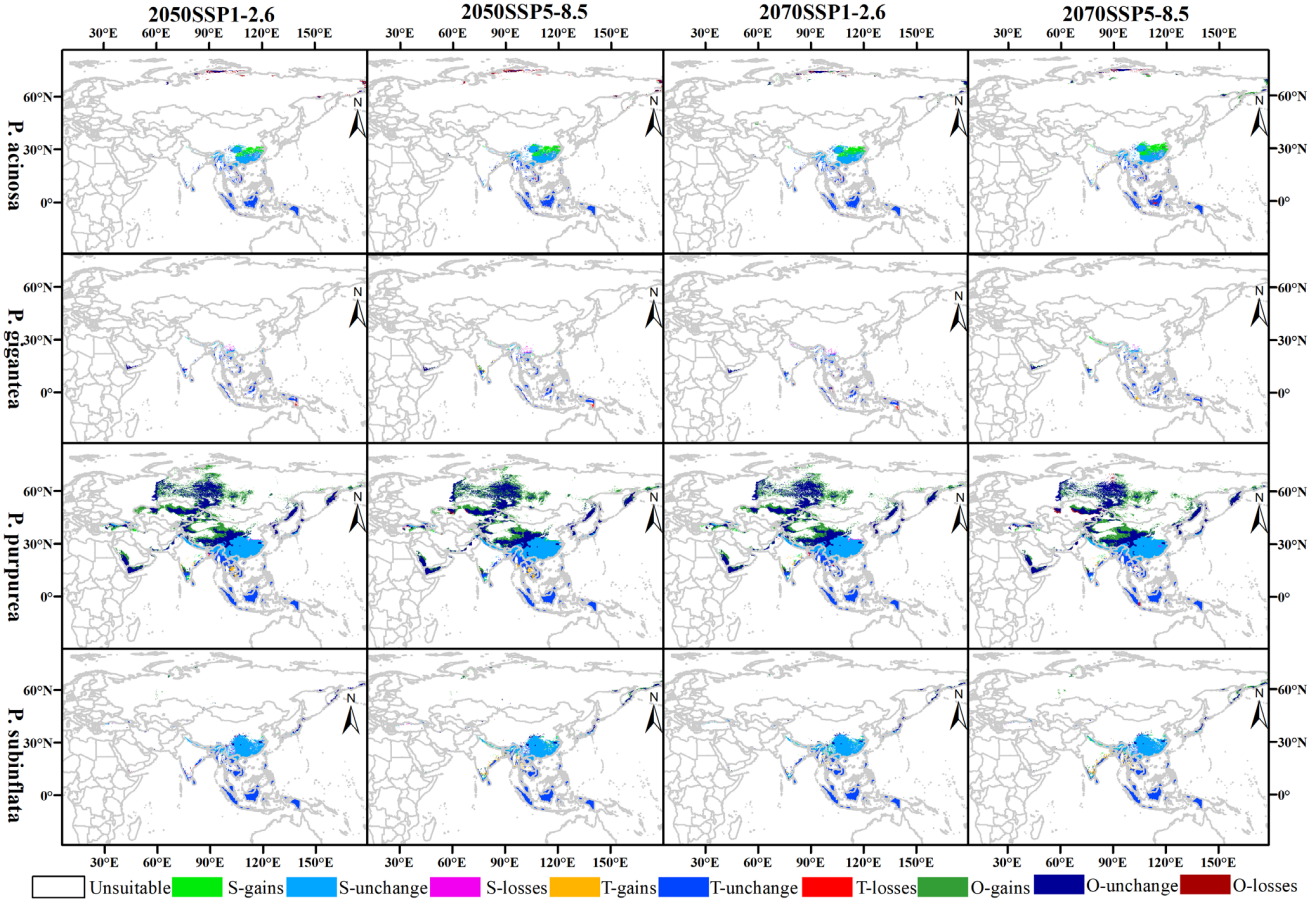
Changes in suitable habitats of four *Pleurozia* species over time relative to the current climate model. S, subtropical; T, tropical; O, other regions; gains, newly suitable areas; unchang, areas with no change in suitability; losses, areas that become unsuitable.

Under future climate scenarios, MaxEnt model projections indicate that the four *Pleurozia* species exhibit distinct distribution dynamics across different time periods and emission pathways (Figures 6 and 7). However, the overall extent of suitable habitats increases for all species (Table S7), with distributions generally tending to expand northward, mirroring the trend observed for the genus‐level suitable areas.

*P. acinosa* exhibited a pronounced trend of northward expansion, particularly under the SSP5‐8.5 scenario, with newly suitable areas emerging mainly in southern China, the eastern coast of India, the western Himalayas, and the Bering Sea coast. However, under the high‐emission scenario, habitat loss by the 2070s is projected to occur primarily in parts of southern China, the Indochina Peninsula, and certain regions of Indonesia.

For *P. gigantea* , suitable habitats are projected to decline notably in Yunnan, China, and on some islands of Southeast Asia under future warming scenarios. In contrast, the greatest increase in total suitable habitat is expected by the 2070s under SSP5‐8.5, particularly in the Himalayas, southern Vietnam, Sumatra, and coastal areas of India.

*P. purpurea* exhibits the most extensive distributional shifts, with significant northward expansion into western China and further into central to northern Russia, westward extension to northern Türkiye, and eastward expansion to the Bering Sea coast. By contrast, slight habitat loss is projected mainly in eastern China, the Indochina Peninsula, Sumatra, central Kazakhstan, and northern Russia, especially by the 2070s under SSP5‐8.5.

*P. subinflata* exhibits relatively limited distributional changes, characterized by slight expansion in the northern Indochina Peninsula, coastal regions of India, eastern China, the Himalayas, and the Bering Sea coast. In contrast, habitat loss is projected in southwestern and southeastern China by the 2070s under SSP5‐8.5, whereas suitable habitats in both regions are projected to increase under SSP1‐2.6 over the same period.

### 3.6 The Distribution Centroid Migration of *Pleurozia* and Four *Pleurozia* Species

**FIGURE 8:**
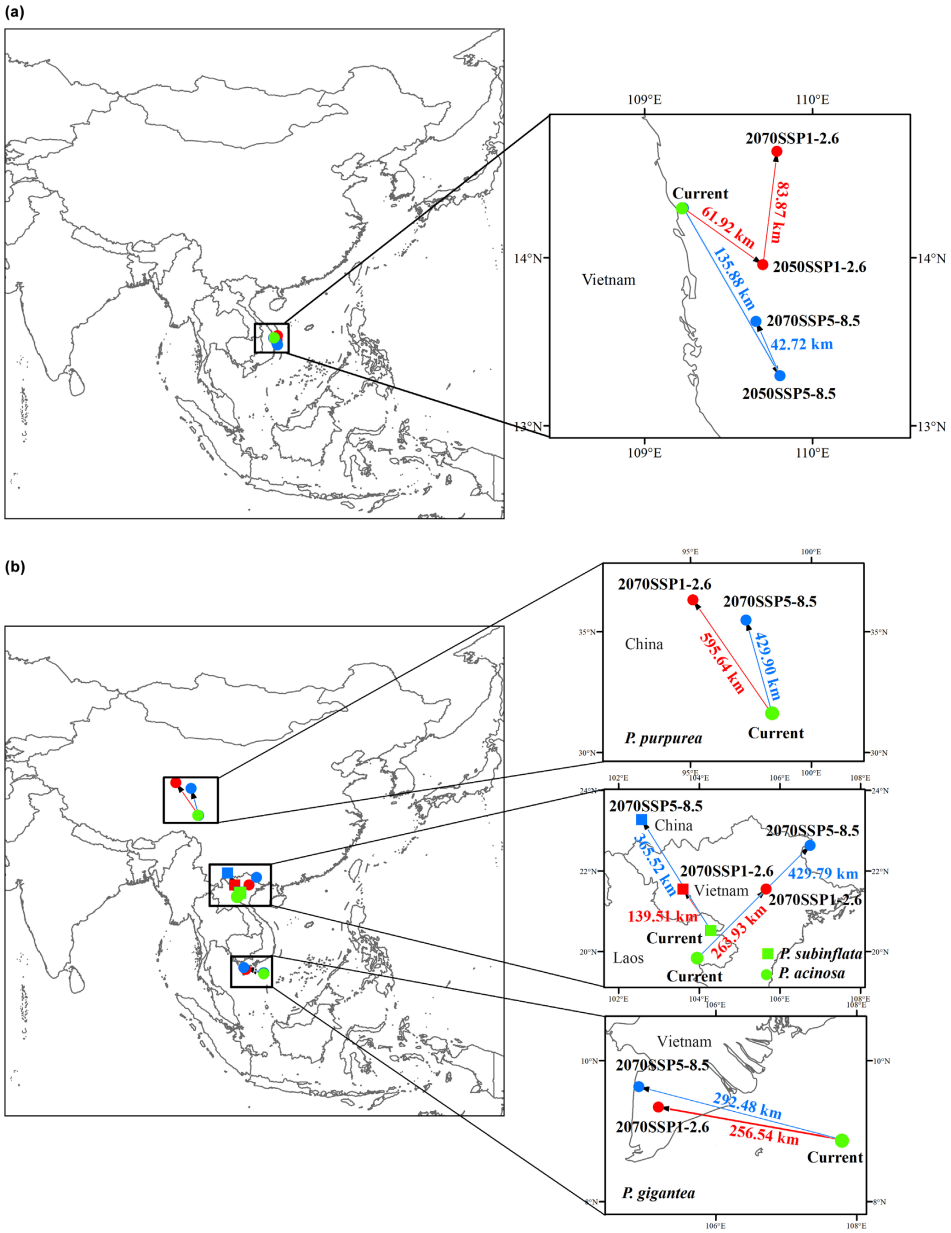
Shifts in the distribution centroid of suitable habitats for (a) *Pleurozia* and (b) four *Pleurozia* species under different climatic scenarios.

Changes in the distribution centroid indicated that, at the genus level, *Pleurozia* exhibited relatively limited migration under future climate scenarios, with an initial southward shift by the 2050s followed by a northward movement by the 2070s. Under the current climate, its centroid was located at 14.30° N, 109.23° E, off the eastern coast of Vietnam. Overall, the centroid showed a net northeastward displacement of 68.04 km under SSP1‐2.6, but a net southeastward displacement of 93.42 km under SSP5‐8.5 (Figure 8a).

Under future climate scenarios, the distribution centroids of the four *Pleurozia* species showed species‐specific shifts by the 2070s, but all exhibited an overall tendency to migrate toward higher latitudes (Figure 8b). The centroids of *P. acinosa* , *P. gigantea* , and *P. subinflata* , currently located in tropical regions, showed greater migration distances under SSP5‐8.5 (292.48–429.79 km) than under SSP1‐2.6 (139.51–263.93 km). In contrast, *P. purpurea* , whose centroid is currently located in the subtropics, exhibited the largest overall displacement, although its migration distance was greater under SSP1‐2.6 (595.64 km) than under SSP5‐8.5 (429.90 km), differing from the pattern observed in the other three species.

## 4 Discussion

Species distribution models (SDMs) are important tools for analyzing the relationships between species and environmental factors. Commonly utilized SDMs include the Bioclimatic Analysis and Prediction System (BIOCLIM), Genetic Algorithm for Rule Set Generation (GARP), and Maximum Entropy Modeling (MaxEnt) (Xu et al. 2023). Among these, MaxEnt modeling is widely applied in studies on the impacts of climate change on species distribution (Subba et al. 2018; Wang et al. 2019), as well as in the management and conservation of endangered species (Li et al. 2013), owing to its advantages such as short processing time, easy operation, strong data‐handling capability, and high predictive accuracy even with a small sample size (Ahmed et al. 2015).

In this study, all seven *Pleurozia* species recorded in Asia were modeled at the genus level, which allowed the inclusion of endemic and narrow‐range species and thus provided a more comprehensive representation of the genus' potential distribution. The genus‐level MaxEnt model showed excellent performance, with a mean test AUC of 0.975 (SD = 0.0009) across replicate runs, indicating high predictive accuracy and reliability. In addition, four species with relatively large populations were modeled individually, and similar predictive performance was obtained at the species level. The moderately and highly suitable habitats of these four species largely overlapped with one another, as well as with the genus‐level suitable area, although *P. purpurea* exhibited a broader potential distribution, mainly due to its greater extent of low‐suitability habitats in temperate regions. Together, these results suggest that genus‐level modeling effectively captures the overall diversity and potential range of *Pleurozia*. Moreover, the predicted distributions under current climatic conditions are broadly consistent with previous studies of *Pleurozia* in Asia (Thiers 1993), further supporting the reliability of the modeled potential distribution at the continental scale.

### 4.1 Environmental Factors

#### 4.1.1 Evergreen Broadleaf Forests

Using MaxEnt modeling, this study identified key environmental variables shaping the distribution of *Pleurozia*. Evergreen broadleaf forest cover (Cons2) was the most important predictor at the genus level, accounting for 40.2% of model contribution, and was also particularly influential for *P. acinosa* (61.1%) and *P. gigantea* (39.8%). Its strong positive relationship with occurrence probability further emphasizes the critical role of evergreen broadleaf forest cover in determining the distribution of *Pleurozia*.

In contrast, leaf area index (Lai) emerged as an important variable for *P. purpurea* and *P. subinflata* , contributing 20.0% and 29.2%, respectively. As an indicator of canopy structure, Lai reflects the role of vegetation in regulating understory microclimate (Ismaeel et al. 2024). Dense canopies can buffer macroclimatic warming by maintaining cooler, more humid, and more stable microenvironments (De Frenne et al. 2013), while also increasing water availability through interception of rainfall and atmospheric moisture (e.g., fog and dew) (Takagi and Shinohara 2025; Zhao et al. 2025) for epiphytic bryophytes. Together with evergreen broadleaf forest cover (Cons2), these results suggest that forest cover and canopy structure jointly promote habitat suitability for *Pleurozia* by enhancing microclimatic buffering and substrate provision (e.g., bark and branches). Consequently, intact evergreen broadleaf forests likely function as important refugia for epiphytic bryophytes under ongoing climate change.

#### 4.1.2 Thermal Variability

Temperature variability was a major determinant of *Pleurozia* distribution at both the genus and species levels. At the genus level, temperature annual range (Bio7) and mean diurnal range (Bio2) contributed strongly to the model. At the species level, although the specific temperature‐related variables differed among species, temperature variability consistently remained the dominant climatic driver: Bio2 was particularly important for *P. acinosa* and *P. subinflata* , Bio7 for *P. purpurea* and *P. subinflata* , and temperature seasonality (Bio4) for *P. gigantea* . The decline in occurrence probability with increasing temperature variability further indicates that *Pleurozia* is generally associated with thermally stable habitats.

Climate change is modifying not only mean temperatures but also the magnitude of thermal variability. For example, tropical regions experienced a warming of approximately 0.39°C and an increase of about 0.3°C in diurnal temperature range between 1975 and 2013 (Wang and Dillon 2014). Such increases in temperature variability may impose additional physiological stress on *Pleurozia* species, potentially restricting their distribution by exceeding their tolerance limits to thermal fluctuations.

These findings are consistent with previous distribution modeling of bryophytes (Deng et al. 2023; Chawengkul et al. 2024) and further underscore the critical role of temperature variation in shaping the persistence and distribution of epiphytic bryophytes. Importantly, the shaded understory of evergreen broadleaf forests may buffer *Pleurozia* against increasing thermal variability.

#### 4.1.3 Altitude and Montane Cloud Forests

Altitude also represents an important environmental factor shaping the distribution of *Pleurozia* at the genus level. At the species level, its influence is even more pronounced for *P. gigantea* and *P. purpurea* , indicating strong elevational constraints on their distributions. Our results indicate that *Pleurozia* is primarily distributed between 1000 and 3500 m, which closely corresponds to the core elevational range of tropical and subtropical montane cloud forests (Bruijnzeel et al. 2011).

Montane cloud forests are humid (sub)tropical ecosystems frequently immersed in clouds and mist, typically occurring on mountain slopes, ridges, and summits at elevations of 500–3500 m (Bubb et al. 2004). These environments are characterized by high humidity, relatively low temperatures, and reduced thermal fluctuations, which together create highly favorable conditions for the growth, reproduction, and persistence of bryophytes (Pócs 1982; Deng et al. 2023). Consequently, such habitats represent key ecological niches for epiphytic bryophytes, including *Pleurozia*.

However, ongoing climate warming is expected to alter and potentially reduce the extent of montane cloud forests through upward shifts, fragmentation, and contraction of suitable habitats. The lack of higher‐elevation refugia leaves these species with nowhere to migrate, turning mountaintop microrefugia into demographic traps that accelerate population decline and eventual extinction (Pertoldi and Bach 2007; Zotz and Bader 2009). Evidence from the Canary Islands further suggests that shifts in the cloud belt, particularly a lowering of its upper limit, can strongly influence bryophyte distribution (Lloret and González‐Mancebo 2011). Together, these findings indicate that changes in cloud forest dynamics may directly affect the distribution and persistence of epiphytic bryophytes.

### 4.2 Changes in Suitable Habitats Under Climate Scenarios

#### 4.2.1 Overall Trends and Regional Patterns in Habitat Change

Under both climate model projections, the suitable habitats of *Pleurozia* and four species have a consistent tendency to expand northward. This pattern is consistent with the general response of species to climate warming, where suitable habitats shift toward higher latitudes and elevations (e.g., Parmesan 2006; Hughes 2011; Sun et al. 2020). The projected range expansion of *Pleurozia* from Southeast Asia toward southern China, westward into the Himalayas, and northeast along the coast to Japan likely reflects the influence of future climate change, where rising temperatures and increased precipitation create warmer, more humid conditions in montane forests (Pepin et al. 2025), thus expanding potential habitats. Notably, the overall increase in suitable area is largely due to the growth of low‐suitability habitats, suggesting that the genus' ecological niche is broadening at the margins rather than experiencing a major increase in highly optimal habitats.

Differences between climate scenarios further highlight *Pleurozia*'s sensitivity to warming intensity. At the genus level, the moderate‐emission SSP1‐2.6 scenario supports the largest extent of suitable habitats, particularly in subtropical regions, likely because moderate warming alleviates low‐temperature constraints while maintaining favorable hydrothermal conditions, avoiding physiological stress associated with excessive heat (Hao et al. 2021). In contrast, under the high‐emission SSP5‐8.5 scenario, total suitable area and low‐ to moderate‐suitability zones decline from 2050 to 2070, while subtropical regions within highly suitable habitats fall below contemporary levels, suggesting that excessive warming may exceed the optimal climatic thresholds for *Pleurozia* in some regions.

Regionally, tropical island regions in equatorial Southeast Asia show relative stability or expansion, likely due to projected increases in temperature and precipitation creating more stable, warm, and humid forest microclimates. Additionally, dense tropical forest canopies can buffer external warming, helping to maintain favorable understory conditions (De Frenne et al. 2021; Hes et al. 2024; Trew et al. 2024). However, some studies suggest that tropical bryophytes are not immune to climate change. For example, rising temperatures have been shown to negatively affect the distribution of the tropical moss *Leucobryum aduncum*, indicating a potential risk of range contraction (Chawengkul et al. 2024). In contrast, subtropical regions exhibit more dynamic changes, likely because species in these climatically transitional areas occur closer to their physiological and climatic tolerance limits and are therefore more sensitive to shifts in temperature and moisture availability, consistent with evidence from species distribution studies under climate change scenarios (Su et al. 2021; Zu et al. 2021). Meanwhile, montane areas, particularly the Himalayas and mountainous regions of Southwest China, may serve as climatic refugia by providing stable and humid microhabitats that buffer species against regional climate change (Tang et al. 2018; Meng et al. 2019).

#### 4.2.2 Species‐Level Variation in Habitat Responses

At the species level, the results show that the suitable habitats of four *Pleurozia* species, particularly the moderate‐ to high‐suitability areas, are mainly distributed in tropical and subtropical regions, with substantial spatial overlap. Nevertheless, they exhibit distinct responses to climate change, reflecting differences in ecological niches and environmental tolerances. *P. acinosa* shows expansion potential, particularly under high‐emission scenarios, but the total suitable habitat in tropical regions is projected to decrease across all scenarios. In contrast, *P. gigantea* , which is mainly restricted to tropical regions, is projected to experience a slight increase in total suitable habitats only under SSP5‐8.5. *P. purpurea* exhibits the most pronounced distributional shifts, suggesting higher dispersal ability or ecological plasticity, whereas *P. subinflata* shows relatively limited changes, reflecting a more conservative ecological strategy.

These contrasting responses can be understood within a niche‐based framework. Bryophyte species often show complex patterns of niche breadth and overlap, and their communities may persist in non‐equilibrium states, leading closely related species to respond differently to climate change (Slack 1990). Species showing positive responses are likely to possess broader climatic niches or greater tolerance to key environmental factors such as temperature and precipitation. In contrast, negatively responding species may occur near their physiological limits or depend more strongly on specific and vulnerable microhabitats, such as high‐altitude cloud forests.

Similar patterns have been reported in other bryophyte studies. For example, projections for 17 *Sphagnum* species in Türkiye indicate divergent future trajectories, with some species remaining stable while others undergoing substantial changes (Abay and Gül 2025a). Likewise, studies on four unisexual bryophyte species have revealed sex‐specific responses to climate change, highlighting the importance of considering interspecific, intraspecific, and even population‐level variations (Martins et al. 2025).

#### 4.2.3 The Distribution Centroid Migration

The projected centroid shifts of *Pleurozia* and its four representative species indicate clear directional changes under future climate scenarios, underscoring the role of climate warming in shaping spatial redistribution patterns. At the genus level, centroid displacement remains relatively limited, suggesting that the distributional center of *Pleurozia* is likely to persist in tropical regions. Centroid migration nevertheless exhibits a consistent two‐stage pattern across climate scenarios, with a southward shift from the present to 2050 followed by a northward shift from 2050 to 2070. This nonlinear pattern suggests a temporally dynamic response to climate change: although intact evergreen broadleaf forests and montane cloud forests may buffer temperature fluctuations and provide climatic refugia for epiphytic bryophytes, the effect of rising temperature becomes increasingly pronounced over time, ultimately driving the centroid toward higher latitudes. Beyond latitudinal shifts, climate warming may also induce upward elevation migration of *Pleurozia* species. Such elevational shifts may represent a potential adaptive strategy that allows these epiphytic bryophytes to track suitable microclimatic conditions and mitigate thermal stress, which could partly explain the relatively limited horizontal displacement of the genus' distribution centroid under future climatic scenarios.

At the species level, centroid shifts are more variable, yet all species exhibit a consistent northward migration under different climate scenarios. Notably, *Pleurozia acinosa* , *P. gigantea* , and *P. subinflata* show longer migration distances under the high‐emission SSP5‐8.5 scenario than under SSP1‐2.6, suggesting that stronger warming may accelerate latitudinal redistribution. Overall, these patterns support a general shift toward higher latitudes under climate warming, consistent with previous studies (e.g., Parmesan 2006; Sun et al. 2020; Ma et al. 2022).

### 4.3 Study Uncertainty, Model Limitations, and Conservation Implications

Our results are subject to several sources of uncertainty. First, the completeness and accuracy of species occurrence records may influence model performance and prediction reliability (Rocchini et al. 2011). Second, we assumed unlimited dispersal, although dispersal constraints may still affect species' ability to track suitable habitats, even for bryophytes with relatively high dispersal capacity (Vanderpoorten et al. 2019). Third, the models considered only abiotic variables, while biotic factors, such as human disturbance, land‐use change, and species interactions, may also play important roles in shaping species distributions (Boulangeat et al. 2012). Finally, although epiphytic bryophytes depend strongly on forest cover and structure that provide suitable microhabitats, our projections assumed constant evergreen broadleaf forest cover over time and did not account for potential future changes, which are already occurring and are likely to continue in the coming decades. Despite these limitations, our findings provide insights into the potential distribution of *Pleurozia* and its representative species under future climate scenarios and offer guidance for the conservation of epiphytic bryophyte diversity in tropical and subtropical rainforests.

Studies have shown that even modest warming has significantly reduced tree growth rates in tropical regions (Way and Oren 2010). In addition to rising temperatures, disturbances such as selective logging, fires, and edge effects substantially contribute to tropical forest degradation (Bourgoin et al. 2024), leading to habitat fragmentation and the loss of suitable microhabitats for epiphytic bryophytes. Together, these pressures compound the impacts of climate warming, placing epiphytic bryophytes at an elevated risk of decline and extinction (He et al. 2016), particularly in high‐elevation cloud forests where habitat loss and fragmentation are most severe (Still et al. 1999; Foster 2001; Bubb et al. 2004). Therefore, conserving the stability and continuity of evergreen broadleaf and cloud forest ecosystems, restoring degraded habitats with native vegetation, and reducing logging and fire disturbances in regions such as southern China, the Himalayas, and the Malay Archipelago are critical for safeguarding *Pleurozia* and other epiphytic bryophytes.

## 5 Conclusion

This study used MaxEnt modeling to identify key environmental drivers of *Pleurozia* distribution in Asia and to project habitat shifts under future climate scenarios. The results highlight that the distribution and future dynamics of *Pleurozia* are shaped by the combined effects of macroclimate and forest‐associated microhabitat buffering. Evergreen broadleaf and montane cloud forests serve as critical habitats, providing both suitable substrates and stable temperature and moisture microclimates that mitigate thermal fluctuations for epiphytic bryophytes.

Although climate warming drives a general tendency for range shifts toward higher latitudes, the relatively limited centroid displacement at the genus level suggests that *Pleurozia* may persist within tropical regions by relying on stable forest environments and microrefugia rather than extensive migration. In contrast, species‐level responses are more heterogeneous, reflecting differences in niche breadth, climatic tolerance, and dispersal capacity, which may lead to divergent distribution patterns under future climate scenarios.

Climate warming generally drives range shifts toward higher latitudes, particularly between the 2050s and 2070s, while the suitable habitat area of *Pleurozia* is projected to increase under all future scenarios, with the largest extent under SSP1‐2.6. Meanwhile, the relatively limited centroid displacement at the genus level suggests that *Pleurozia* may persist in tropical regions through stable forest environments and microrefugia rather than extensive migration. In contrast, species‐level responses are more heterogeneous in spatial change, suitable habitat area, and migration distance, indicating divergent distribution dynamics under future climate scenarios that likely reflect differences in niche breadth, climatic tolerance, and dispersal capacity.

Overall, the vulnerability and persistence of epiphytic bryophytes under climate change depend not only on climatic suitability but also on the integrity and continuity of forest ecosystems. In particular, the conservation of evergreen broadleaf and montane cloud forests will be essential for maintaining microclimatic buffering capacity and supporting bryophyte diversity. Integrating climate‐driven projections with forest conservation and management strategies is therefore crucial for sustaining tropical and subtropical biodiversity in a warming world.

## Author Contributions

**Liangtao Huang:** conceptualization (lead), data curation (lead), formal analysis (lead), investigation (lead), methodology (lead), visualization (lead), writing – original draft (lead), writing – review and editing (lead). **Feihan Chen:** data curation (equal), formal analysis (equal), investigation (equal), writing – original draft (equal), writing – review and editing (equal). **Yuwei Su:** data curation (equal), formal analysis (equal), methodology (equal), supervision (equal), writing – review and editing (equal). **Zhongyi Sun:** conceptualization (equal), data curation (equal), methodology (equal), supervision (lead), writing – review and editing (equal). **Xiao Huang:** conceptualization (equal), methodology (equal), supervision (equal), writing – review and editing (equal). **Huimin Lin:** data curation (equal), investigation (equal), writing – review and editing (equal). **De Gao:** data curation (equal). **Lina Zhang:** conceptualization (lead), funding acquisition (lead), investigation (lead), methodology (lead), supervision (lead), writing – original draft (lead), writing – review and editing (lead).

## Funding

This study was funded by the National Natural Science Foundation of China (Grant Nos. 32160315, 31760054, 42271045).

## Conflicts of Interest

The authors declare no conflicts of interest.

## Supporting information


**Appendix S1:** Details on species occurrence data, environmental variable screening, and MaxEnt modeling results, including Tables S1–S7 and Figures S1–S9.
**Table S1:** 81 valid data points of occurrence used for modeling of this study.
**Table S2:** Screened environmental variables.
**Table S3:** Optimal parameters of MaxEnt model for *Pleurozia* and four *Pleurozia* species.
**Table S4:** Dominant environmental variables influencing geographic distribution patterns of *Pleurozia*.
**Table S5:** Dominant environmental variables influencing geographic distribution patterns of four *pleurozia* species.
**Table S6:** Changes in the areas (× 10^4^ km^2^) of suitable habitats for *Pleurozia* under the current and future climatic conditions. “Total” is the total area of suitable habitats.
**Table S7:** Changes in the areas (× 10^4^ km^2^) of suitable habitats for four *Pleurozia* species under the current and future climatic conditions. “Total” is the total area of suitable habitats.
**Figure S1:** Heat map of environmental variable correlations.
**Figure S2:** AUC values of *Pleurozia* and four *Pleurozia* species. Note: A: *Pleurozia*; B: *P. subinflata* ; C: *P. acinosa* ; D: *P. gigantea* ; E: *P. purpurea* .
**Figure S3:** The Jackknife test results of environmental variables for *Pleurozia* in Asia. Note: The dark blue bar indicates the gain obtained by using each variable individually, the light blue bar indicates the gain lost by removing a single variable from the whole model, and the red bar indicates the gain obtained by using all variables.
**Figure S4:** Response curves of four environmental factors.
**Figure S5:** The Jackknife test results of environmental variables for four *pleurozia* species in Asia.
**Figure S6:** Response of *P. acinosa* to the three key environmental variables.
**Figure S7:** Response of *P. gigantea* to the three key environmental variables.
**Figure S8:** Response of *P. purpurea* to the four key environmental variables.
**Figure S9:** Response of *P. subinflata* to the three key environmental variables.

## Data Availability

The data are provided in the Supporting Information for this article.
