# Supplementary material for: Tree Cover and Temperature Shape the Distribution of Epiphytic Pleurozia in Asia: Forest Havens in a Warming Climate
Source: Ecol Evol. 2026 Jun 1;16(6):e73657. doi: 10.1002/ece3.73657 (PMC13238694; doi:10.1002/ece3.73657)

**Tree Cover and Temperature Shape the Distribution of Epiphytic *Pleurozia* in Asia: Forest Havens in a Warming Climate**

Liangtao Huang, Feihan Chen, Yuwei Su, Zhongyi Sun, Xiao Huang, Huimin Lin, De Gao, Lina Zhang*

**SUPPORTING INFORMATION**

**Appendix S1.** Details on species occurrence data, environmental variable screening, and MaxEnt modelling results, including Tables S1–S7 and Figures S1–S9.

**Table S1** 81 valid data points of occurrence used for modelling of this study.

| Data point order | Species | Latitude | Longitude |
| --- | --- | --- | --- |
| 1 | *Pleurozia acinosa* | 4.479166667 | 101.3958333 |
| 2 | *Pleurozia acinosa* | 3.4375 | 101.7708333 |
| 3 | *Pleurozia acinosa* | 21.4375 | 105.6458333 |
| 4 | *Pleurozia acinosa* | 21.47916667 | 105.6458333 |
| 5 | *Pleurozia acinosa* | 22.60416667 | 105.8541667 |
| 6 | *Pleurozia acinosa* | 12.1875 | 108.7291667 |
| 7 | *Pleurozia acinosa* | 19.1875 | 109.7708333 |
| 8 | *Pleurozia acinosa* | 18.72916667 | 109.8541667 |
| 9 | *Pleurozia acinosa* | 2.5625 | 112.9791667 |
| 10 | *Pleurozia acinosa* | 6.020833334 | 116.5625 |
| 11 | *Pleurozia acinosa* | -1.479166666 | 120.1875 |
| 12 | *Pleurozia acinosa* | 24.97916667 | 121.3125 |
| 13 | *Pleurozia acinosa* | 24.5625 | 121.3958333 |
| 14 | *Pleurozia acinosa* | 24.6875 | 121.5208333 |
| 15 | *Pleurozia acinosa* | 0.979166667 | 121.6875 |
| 16 | *Pleurozia acinosa* | 30.35416667 | 130.4791667 |
| 17 | *Pleurozia acinosa* | 30.27083333 | 130.5625 |
| 18 | *Pleurozia acinosa* | 30.3125 | 130.5625 |
| 19 | *Pleurozia acinosa* | 30.35416667 | 130.5625 |
| 20 | *Pleurozia acinosa* | 30.3125 | 130.6041667 |
| 21 | *Pleurozia acinosa* | 30.35416667 | 130.6041667 |
| 22 | *Pleurozia acinosa* | 6.8125 | 80.47916667 |
| 23 | *Pleurozia acinosa* | 8.479166667 | 99.72916667 |
| 24 | *Pleurozia articulata* | 6.016667 | 116.533333 |
| 25 | *Pleurozia articulata* | 18.801747 | 109.870706 |
| 26 | *Pleurozia conchifolia var. conchifolia* | -0.395833333 | 100.3125 |
| 27 | *Pleurozia conchifolia var. papillosa* | -1.520833333 | 132.4791667 |
| 28 | *Pleurozia gigantea* | 4.479166667 | 101.3541667 |
| 29 | *Pleurozia gigantea* | 14.47916667 | 101.4791667 |
| 30 | *Pleurozia gigantea* | 17.27083333 | 101.5208333 |
| 31 | *Pleurozia gigantea* | 17.10416667 | 101.6041667 |
| 32 | *Pleurozia gigantea* | 11.89583333 | 103.6875 |
| 33 | *Pleurozia gigantea* | 22.35416667 | 103.8125 |
| 34 | *Pleurozia gigantea* | -6.729166666 | 107.0208333 |
| 35 | *Pleurozia gigantea* | 18.89583333 | 109.6875 |
| 36 | *Pleurozia gigantea* | 3.3125 | 114.1458333 |
| 37 | *Pleurozia gigantea* | 24.5625 | 114.4375 |
| 38 | *Pleurozia gigantea* | 4.0625 | 114.9375 |
| 39 | *Pleurozia gigantea* | 6.0625 | 116.5625 |
| 40 | *Pleurozia gigantea* | -1.020833333 | 119.9791667 |
| 41 | *Pleurozia gigantea* | 16.5625 | 120.8125 |
| 42 | *Pleurozia gigantea* | 16.89583333 | 120.8541667 |
| 43 | *Pleurozia gigantea* | 22.85416667 | 120.8541667 |
| 44 | *Pleurozia gigantea* | 16.60416667 | 120.8958333 |
| 45 | *Pleurozia gigantea* | 13.27083333 | 120.9791667 |
| 46 | *Pleurozia gigantea* | 14.0625 | 121.4791667 |
| 47 | *Pleurozia gigantea* | 9.229166667 | 123.1041667 |
| 48 | *Pleurozia gigantea* | 9.229166667 | 123.1458333 |
| 49 | *Pleurozia gigantea* | 9.3125 | 123.1458333 |
| 50 | *Pleurozia gigantea* | 9.354166667 | 123.1458333 |
| 51 | *Pleurozia gigantea* | 9.229166667 | 123.1875 |
| 52 | *Pleurozia gigantea* | 10.77083333 | 123.2291667 |
| 53 | *Pleurozia gigantea* | 6.979166667 | 125.2708333 |
| 54 | *Pleurozia gigantea* | -1.354166666 | 133.9791667 |
| 55 | *Pleurozia gigantea* | 6.8125 | 80.72916667 |
| 56 | *Pleurozia gigantea* | 7.020833334 | 80.77083333 |
| 57 | *Pleurozia gigantea* | 18.60416667 | 98.47916667 |
| 58 | *Pleurozia johannis-winkleri* | 5.562408 | 116.494405 |
| 59 | *Pleurozia purpurea* | 26.0625 | 102.8541667 |
| 60 | *Pleurozia purpurea* | 22.64583333 | 120.7708333 |
| 61 | *Pleurozia purpurea* | 35.97916667 | 137.9791667 |
| 62 | *Pleurozia purpurea* | 36.0625 | 138.3125 |
| 63 | *Pleurozia purpurea* | 36.85416667 | 138.8958333 |
| 64 | *Pleurozia purpurea* | 36.85416667 | 138.9375 |
| 65 | *Pleurozia purpurea* | 36.52083333 | 138.9791667 |
| 66 | *Pleurozia purpurea* | 27.02083333 | 88.0625 |
| 67 | *Pleurozia purpurea* | 27.4375 | 90.47916667 |
| 68 | *Pleurozia purpurea* | 26.64583333 | 99.72916667 |
| 69 | *Pleurozia subinflata* | 22.3125 | 103.7708333 |
| 70 | *Pleurozia subinflata* | 18.89583333 | 108.6875 |
| 71 | *Pleurozia subinflata* | 18.72916667 | 108.8958333 |
| 72 | *Pleurozia subinflata* | 25.85416667 | 110.4375 |
| 73 | *Pleurozia subinflata* | 2.4375 | 113.4791667 |
| 74 | *Pleurozia subinflata* | 25.35416667 | 116.8125 |
| 75 | *Pleurozia subinflata* | 27.8125 | 117.7291667 |
| 76 | *Pleurozia subinflata* | 22.39583333 | 120.7708333 |
| 77 | *Pleurozia subinflata* | 24.52083333 | 121.6041667 |
| 78 | *Pleurozia subinflata* | 30.3125 | 130.5208333 |
| 79 | *Pleurozia subinflata* | 30.35416667 | 130.5208333 |
| 80 | *Pleurozia subinflata* | 7.604166667 | 80.6875 |
| 81 | *Pleurozia subinflata* | 18.5625 | 98.47916667 |

**Table S2** Screened environmental variables.

| **Code** | **Environmental variable** | **Unit** |
| --- | --- | --- |
| Bio1 | Annual mean temperature | °C |
| Bio2 | Mean diurnal range (Mean of monthly (max. temp – min. temp)) | °C |
| Bio3 | Isothermality (Bio2/Bio7) (×100) | Dimensionless (ratio ×100) |
| Bio4 | Temperature seasonality (standard deviation ×100) | Dimensionless (standard deviation ×100) |
| Bio5 | Max. temperature of warmest month | °C |
| Bio6 | Min. temperature of coldest month | °C |
| Bio7 | Temperature annual range (Bio5-Bio6) | °C |
| Bio8 | Mean temperature of wettest quarter | °C |
| Bio9 | Mean temperature of driest quarter | °C |
| Bio10 | Mean temperature of warmest quarter | °C |
| Bio11 | Mean temperature of coldest quarter | °C |
| Bio12 | Annual precipitation | mm |
| Bio13 | Precipitation of wettest month | mm |
| Bio14 | Precipitation of driest month | mm |
| Bio15 | Precipitation seasonality (Coefficient of variation) | Dimensionless (coefficient of variation) |
| Bio16 | Precipitation of wettest quarter | mm |
| Bio17 | Precipitation of driest quarter | mm |
| Bio18 | Precipitation of warmest quarter | mm |
| Bio19 | Precipitation of coldest quarter | mm |
| Cons1 | Evergreen/Deciduous needleleaf trees | Dimensionless (proportion / percentage) |
| Cons2 | Evergreen broadleaf trees | Dimensionless (proportion / percentage) |
| Cons3 | Deciduous broadleaf trees | Dimensionless (proportion / percentage) |
| Cons4 | Mixed/Other trees | Dimensionless (proportion / percentage) |
| Cons5 | Shrubs | Dimensionless (proportion / percentage) |
| Alt | Altitude | m (meters above sea level) |
| Sr | Solar radiation | kJ m^-2^day^-1^ |
| Wvp | Water vapor pressure | kPa |
| Lai | Leaf area index | Dimensionless (m^2^m^-2^) |

**Table S3** Optimal parameters of MaxEnt model for *Pleurozia* and four *Pleurozia* species*.*

|  | **Type** | **RM** | **FC** | **delta.AICc** |
| --- | --- | --- | --- | --- |
| *Pleurozia* | Default | 1.0 | LQPH | 224.0588 |
|  | Optimization | 1.3 | LQ | 0 |
| *P. acinosa* | Optimization | 2.1 | T | 0 |
| *P. gigantea* | Optimization | 3.1 | LH | 0 |
| *P. purpurea* | Optimization | 1.7 | LP | 0 |
| *P. subinflata* | Optimization | 1.6 | LQP | 0 |

Note: RM: regularization multiplier; FC: feature combination; L: linear; Q: quadratic; P: product; H: hinge; AICc: Akaike Information Criterion; delta.AICc: Differences between AICc values of different models, a smaller delta. AICc suggests a higher probability that the corresponding model is the best fit.

**Table S4** Dominant environmental variables influencing geographic distribution patterns of *Pleurozia.*

| **Code** | **Environmental variable** | **Pc (%)** | **Suitable range** |
| --- | --- | --- | --- |
| Cons2 | Evergreen broadleaf trees | 40.2 | 34.40%–100% |
| Bio7 | Temperature annual range | 23.4 | 1.39℃–19.21℃ |
| Bio2 | Mean diurnal range | 14.2 | 3.61℃–8.01℃ |
| Alt | Altitude | 13.2 | 889.67m–3384.98m |

**Table S5** Dominant environmental variables influencing geographic distribution patterns of four *pleurozia* species.

| **Code** | **Environmental variable** | **Pc (%) and suitable range** | | | |
| --- | --- | --- | --- | --- | --- |
|  |  | *P. acinosa* | *P. gigantea* | *P. purpurea* | *P. subinflata* |
| Cons2 | Evergreen broadleaf trees | 61.1  (25.52%–100%) | 39.8  (40.16%–100%) |  |  |
| Bio2 | Mean diurnal range | 25.9  (3.80℃–8.85℃) |  |  | 31.0  (3.60℃–8.28℃) |
| Bio7 | Temperature annual range |  |  | 20.6  (0.89℃–34.40℃) | 33.6  (1.65℃–22.95℃) |
| Alt | Altitude |  | 17.8  (837.39 m–4960.01 m) | 25.5  (1008.31 m–6793.90 m) |  |
| Lai | Leaf area index |  |  | 20.0  (1.95–7.67) | 29.2  (3.34–7.39) |
| Bio4 | Temperature seasonality (standard deviation ×100) |  | 39.1  (1.71–193.20) |  |  |
| Bio11 | Mean temperature of coldest quarter | 10.3  (6.52℃–35.04℃) |  |  |  |
| Bio18 | Precipitation of warmest quarter |  |  | 11.2  (358.66 mm–3053.60 mm) |  |

Note: Only environmental factors contributing >10.0% individually were selected.

**Table S6** Changes in the areas (× 10^4^ km^2^) of suitable habitats for *Pleurozia* under the current and future climatic conditions. “Total” is the total area of suitable habitats.

| **Period** | **Suitable** | | | **Low-suitable** | | | **Moderate-suitable** | | | **High-suitable** | | |
| --- | --- | --- | --- | --- | --- | --- | --- | --- | --- | --- | --- | --- |
|  | **Total** | **Tropical** | **Sub-tropical** | **Total** | **Tropical** | **Sub-tropical** | **Total** | **Tropical** | **Sub-tropical** | **Total** | **Tropical** | **Sub-tropical** |
| Current | 187.89 | 105.11 | 74.32 | 123.21 | 64.29 | 52.36 | 44.55 | 26.44 | 16.38 | 20.14 | 14.37 | 5.59 |
| 2050SSP1-2.6 | 205.79 | 115.09 | 81.11 | 136.31 | 71.32 | 57.65 | 47.26 | 27.57 | 17.93 | 21.93 | 16.20 | 5.53 |
| 2070SSP1-2.6 | 214.44 | 113.74 | 89.19 | 142.68 | 72.46 | 61.29 | 50.50 | 27.67 | 20.58 | 21.26 | 13.60 | 7.31 |
| 2050SSP5-8.5 | 208.86 | 122.21 | 76.93 | 140.69 | 76.96 | 55.76 | 46.82 | 28.78 | 16.44 | 21.34 | 16.47 | 4.74 |
| 2070SSP5-8.5 | 204.33 | 118.36 | 76.30 | 134.45 | 71.44 | 54.81 | 45.20 | 27.32 | 16.53 | 24.68 | 19.60 | 4.96 |

**Table S7** Changes in the areas (× 10^4^ km^2^) of suitable habitats for four *Pleurozia* species under the current and future climatic conditions. “Total” is the total area of suitable habitats.

| **Species** | **Period** | **Suitable** | | | **Low-suitable** | | | **Moderate-suitable** | | | **High-suitable** | | |
| --- | --- | --- | --- | --- | --- | --- | --- | --- | --- | --- | --- | --- | --- |
|  |  | **Total** | **Tropical** | **Sub-tropical** | **Total** | **Tropical** | **Sub-tropical** | **Total** | **Tropical** | **Sub-tropical** | **Total** | **Tropical** | **Sub-tropical** |
| *P. acinosa* | Current | 389.10 | 211.24 | 121.77 | 161.38 | 43.89 | 63.23 | 86.64 | 53.50 | 32.51 | 141.07 | 113.84 | 27.02 |
|  | 2050SSP1-2.6 | 400.09 | 209.70 | 156.94 | 179.88 | 47.02 | 100.29 | 57.49 | 30.09 | 27.08 | 162.71 | 132.59 | 29.57 |
|  | 2070SSP1-2.6 | 437.12 | 205.45 | 167.86 | 200.51 | 43.10 | 94.69 | 74.87 | 31.24 | 43.09 | 161.74 | 131.12 | 30.08 |
|  | 2050SSP5-8.5 | 396.24 | 206.15 | 166.71 | 187.51 | 51.47 | 113.64 | 42.51 | 20.19 | 21.88 | 166.22 | 134.49 | 31.20 |
|  | 2070SSP5-8.5 | 460.15 | 206.71 | 179.66 | 248.55 | 50.59 | 125.36 | 81.00 | 57.48 | 22.87 | 130.61 | 98.65 | 31.43 |
| *P. gigantea* | Current | 148.09 | 116.89 | 24.92 | 92.52 | 65.88 | 20.61 | 28.88 | 25.95 | 2.69 | 26.69 | 25.06 | 1.62 |
|  | 2050SSP1-2.6 | 129.62 | 101.03 | 22.60 | 70.92 | 46.98 | 18.09 | 28.32 | 25.39 | 2.78 | 30.38 | 28.65 | 1.73 |
|  | 2070SSP1-2.6 | 123.90 | 97.07 | 19.57 | 65.22 | 42.24 | 16.00 | 26.05 | 23.87 | 1.92 | 32.62 | 30.96 | 1.65 |
|  | 2050SSP5-8.5 | 144.37 | 111.89 | 23.64 | 77.78 | 50.20 | 19.15 | 33.68 | 30.68 | 2.61 | 32.90 | 31.01 | 1.88 |
|  | 2070SSP5-8.5 | 178.30 | 137.30 | 31.10 | 115.12 | 81.35 | 25.38 | 33.85 | 28.87 | 3.86 | 28.93 | 27.08 | 1.85 |
| *P. purpurea* | Current | 1083.09 | 261.08 | 287.59 | 706.91 | 157.47 | 96.60 | 220.44 | 67.84 | 88.62 | 155.74 | 35.77 | 102.37 |
|  | 2050SSP1-2.6 | 1564.70 | 284.64 | 295.29 | 1073.27 | 169.09 | 104.88 | 295.55 | 74.65 | 80.53 | 195.88 | 40.90 | 109.88 |
|  | 2070SSP1-2.6 | 1505.08 | 272.25 | 292.94 | 1030.66 | 166.49 | 103.98 | 287.33 | 67.52 | 79.52 | 187.08 | 38.23 | 109.45 |
|  | 2050SSP5-8.5 | 1521.71 | 285.66 | 296.88 | 1043.67 | 172.13 | 95.32 | 288.45 | 73.68 | 91.10 | 189.58 | 39.85 | 110.47 |
|  | 2070SSP5-8.5 | 1423.54 | 261.57 | 293.21 | 911.91 | 162.66 | 89.84 | 289.19 | 62.20 | 76.77 | 222.44 | 36.72 | 126.60 |
| *P. subinflata* | Current | 560.34 | 266.91 | 222.11 | 240.38 | 73.48 | 108.20 | 222.52 | 119.84 | 92.12 | 97.44 | 73.59 | 21.80 |
|  | 2050SSP1-2.6 | 604.68 | 268.62 | 259.29 | 250.35 | 80.29 | 107.23 | 222.08 | 113.02 | 102.19 | 127.25 | 75.31 | 49.87 |
|  | 2070SSP1-2.6 | 595.33 | 281.20 | 231.12 | 266.27 | 76.93 | 119.46 | 227.59 | 125.53 | 90.57 | 101.48 | 78.74 | 21.08 |
|  | 2050SSP5-8.5 | 614.36 | 288.07 | 230.00 | 270.53 | 83.71 | 110.66 | 230.37 | 121.18 | 91.49 | 113.46 | 83.19 | 27.85 |
|  | 2070SSP5-8.5 | 636.76 | 293.39 | 232.80 | 292.13 | 88.34 | 118.46 | 234.59 | 123.71 | 88.16 | 110.04 | 81.34 | 26.18 |

**Figure S1** Heat map of environmental variable correlations.


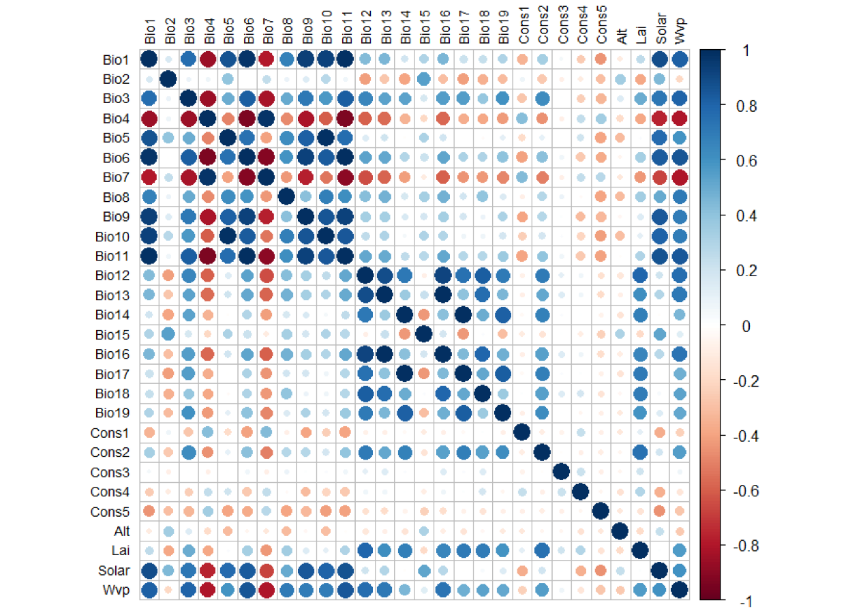


**Figure S2** AUC values of *Pleurozia* and four *Pleurozia* species*.* Note: A: *Pleurozia*; B: *P. subinflata*; C: *P. acinosa*; D: *P. gigantea*; E: *P. purpurea*.


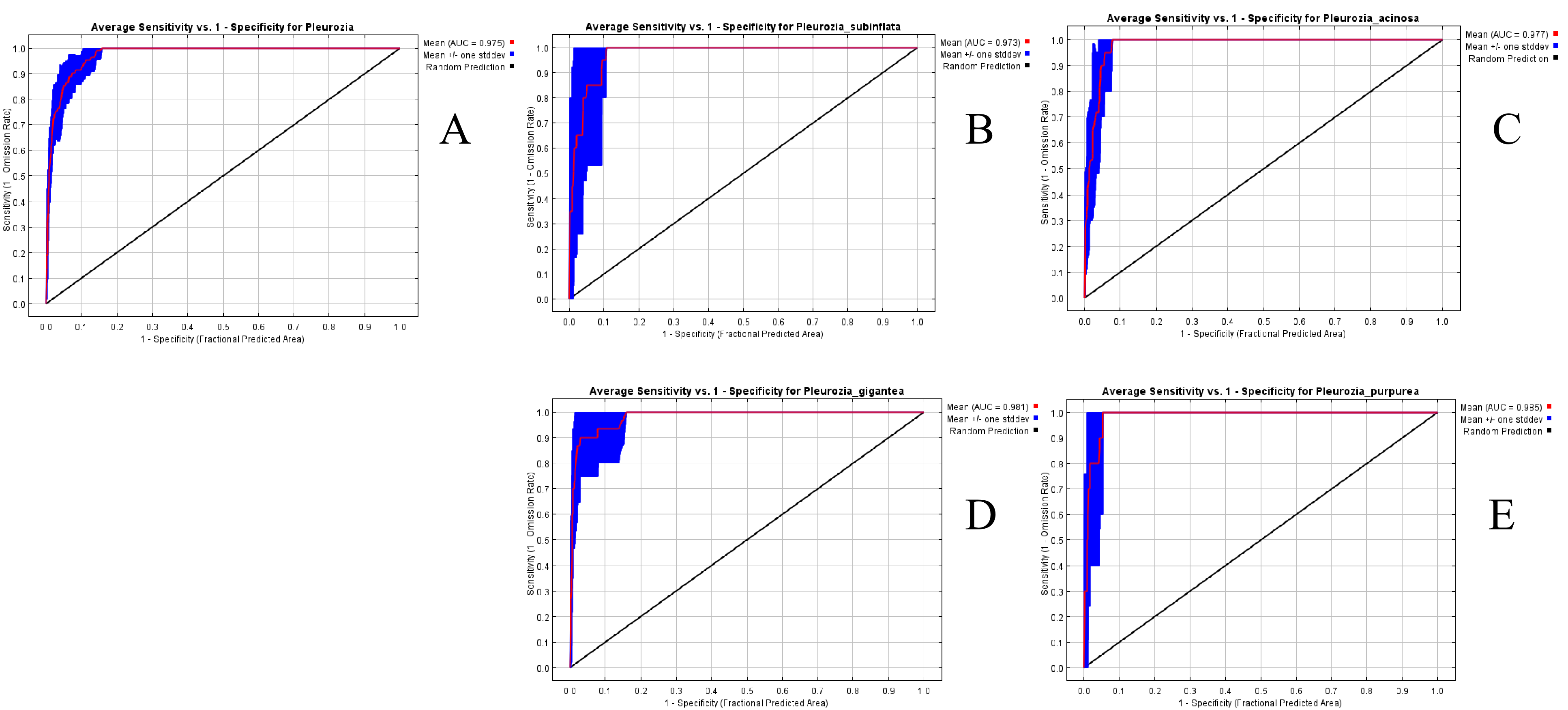


**Figure S3** The Jackknife test results of environmental variables for *Pleurozia* in Asia. Note: The dark blue bar indicates the gain obtained by using each variable individually, the light blue bar indicates the gain lost by removing a single variable from the whole model, and the red bar indicates the gain obtained by using all variables.


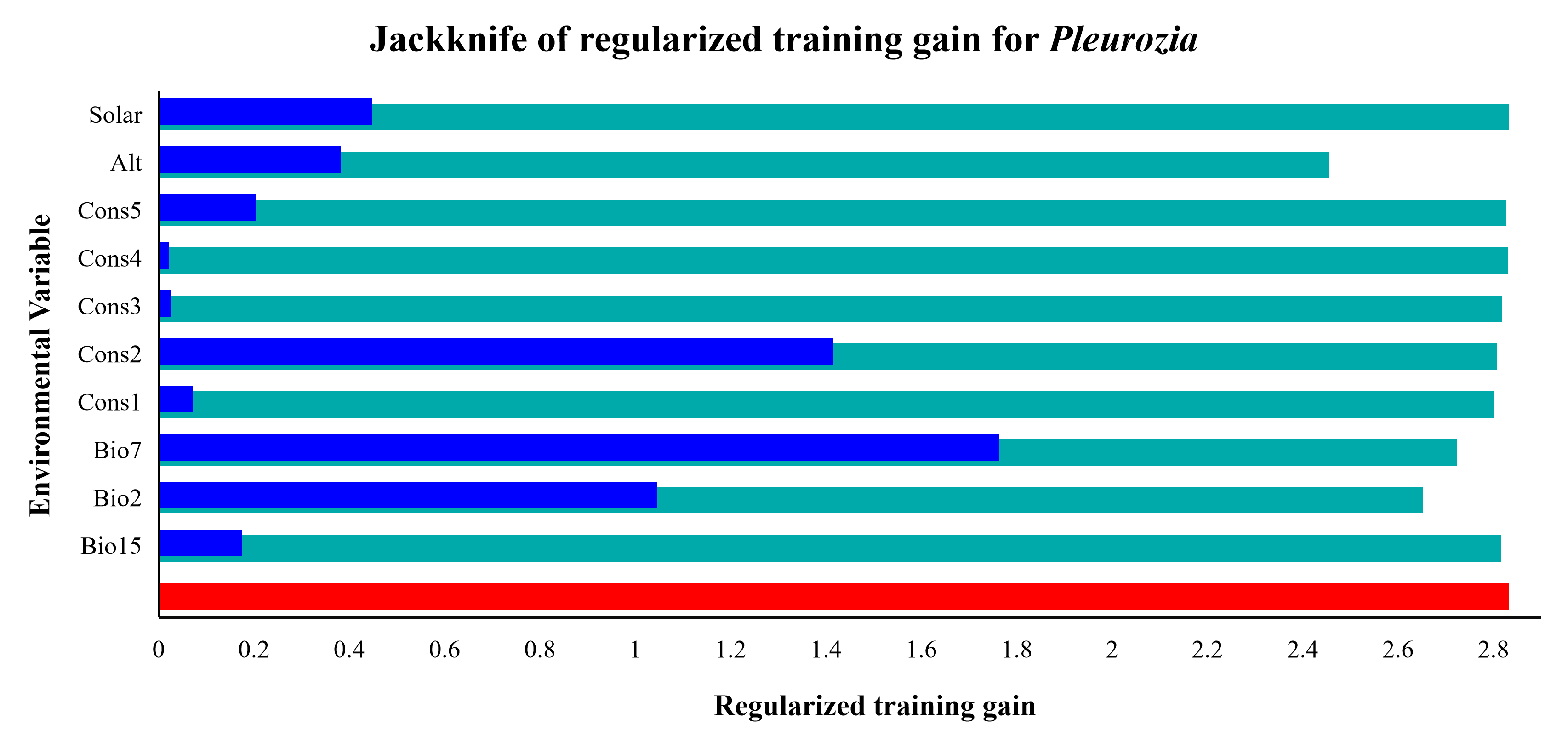


**Figure S4** Response curves of four environmental factors.


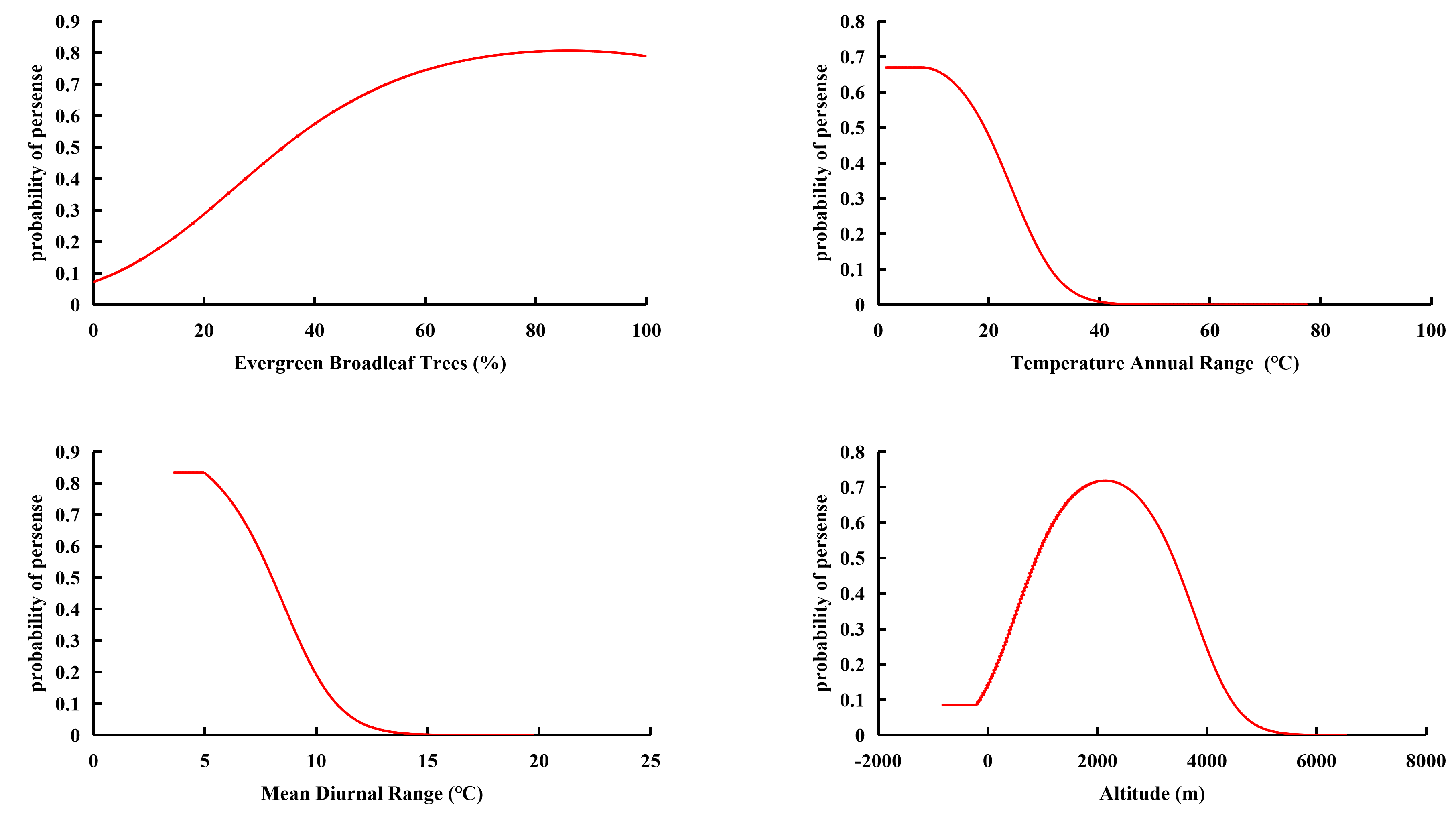


**Figure S5** The Jackknife test results of environmental variables for four *pleurozia* species in Asia.


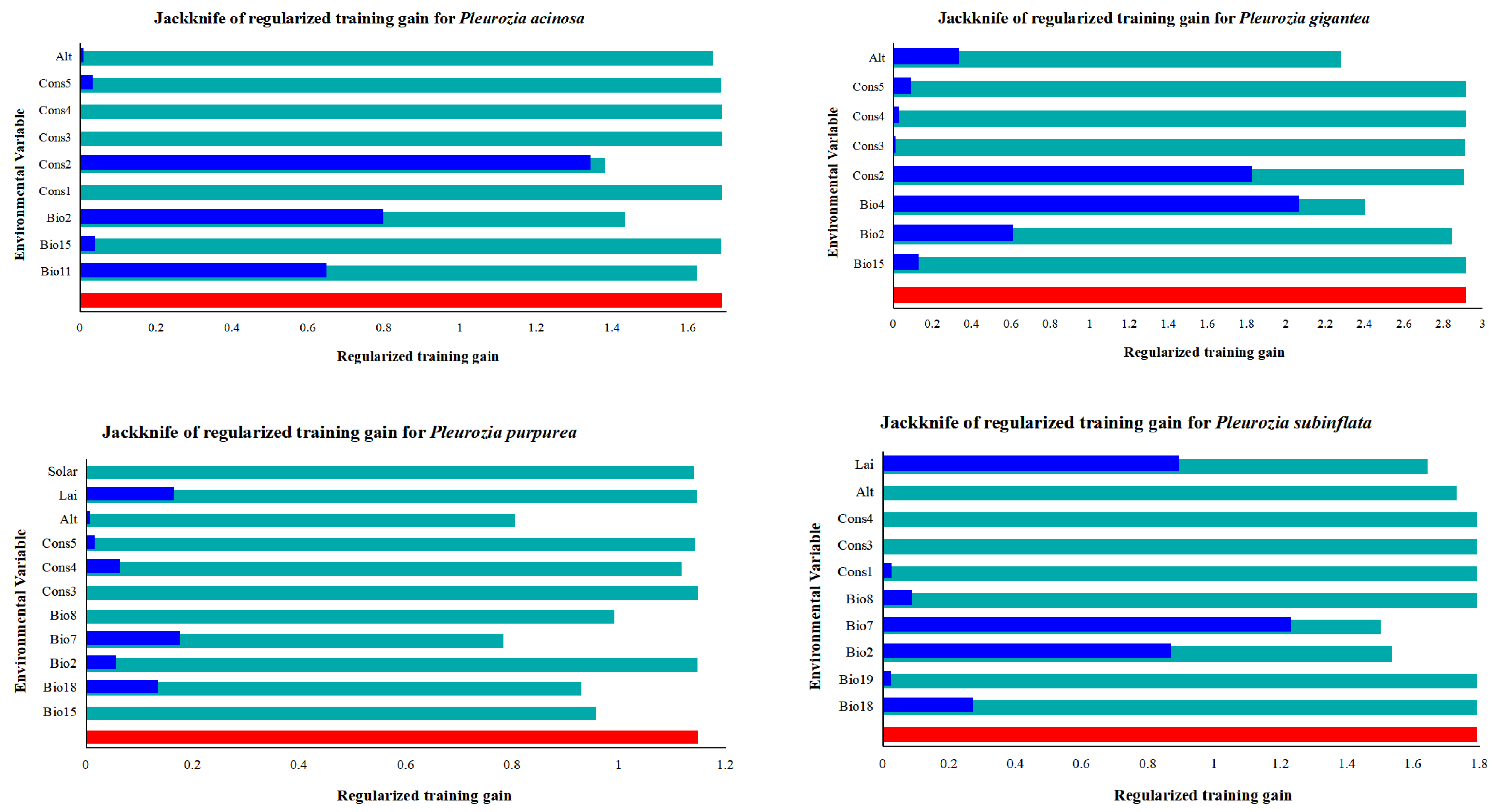


**Figure S6** Response of *P. acinosa* to the three key environmental variables.


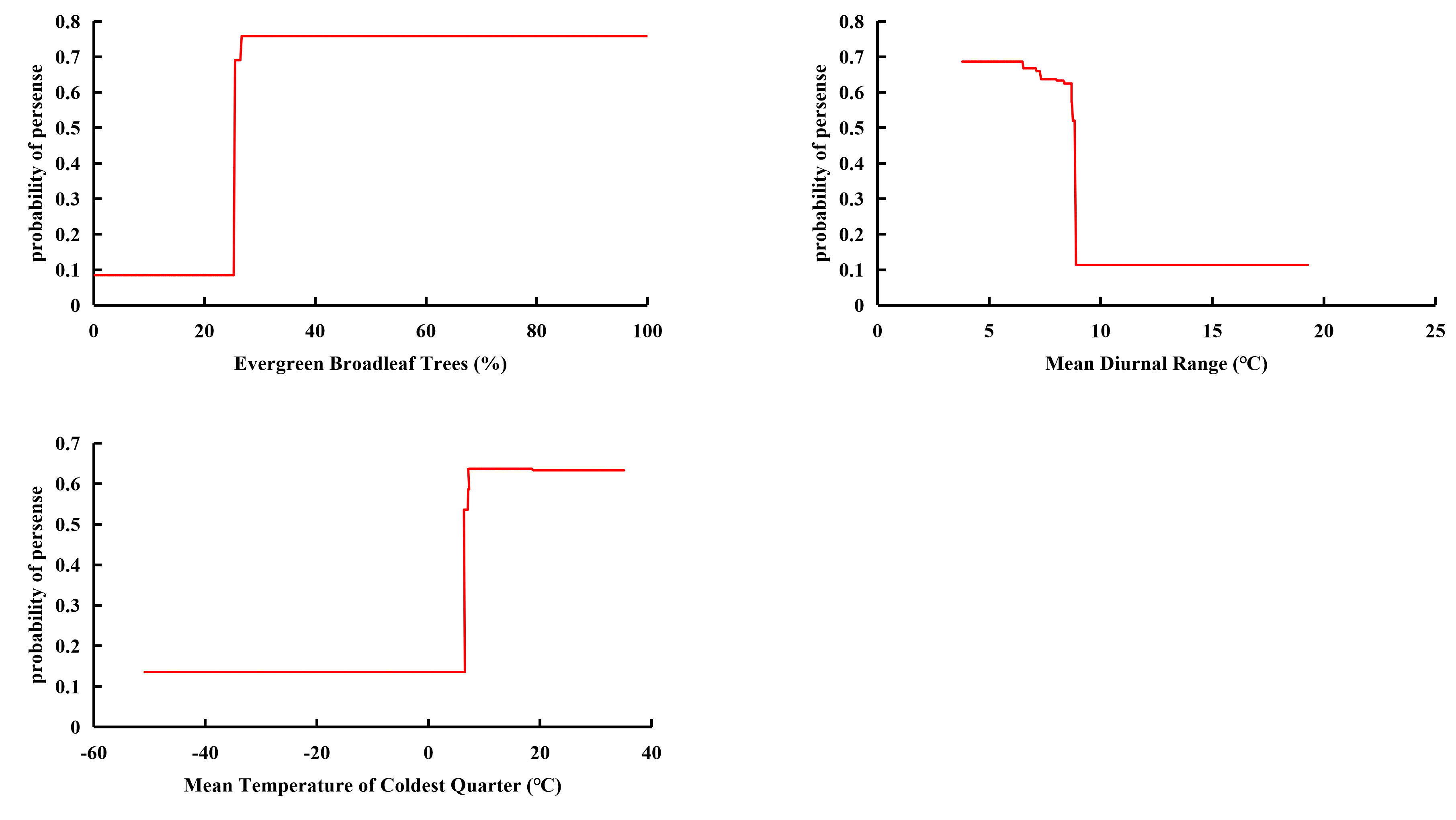


**Figure S7** Response of *P. gigantea* to the three key environmental variables.


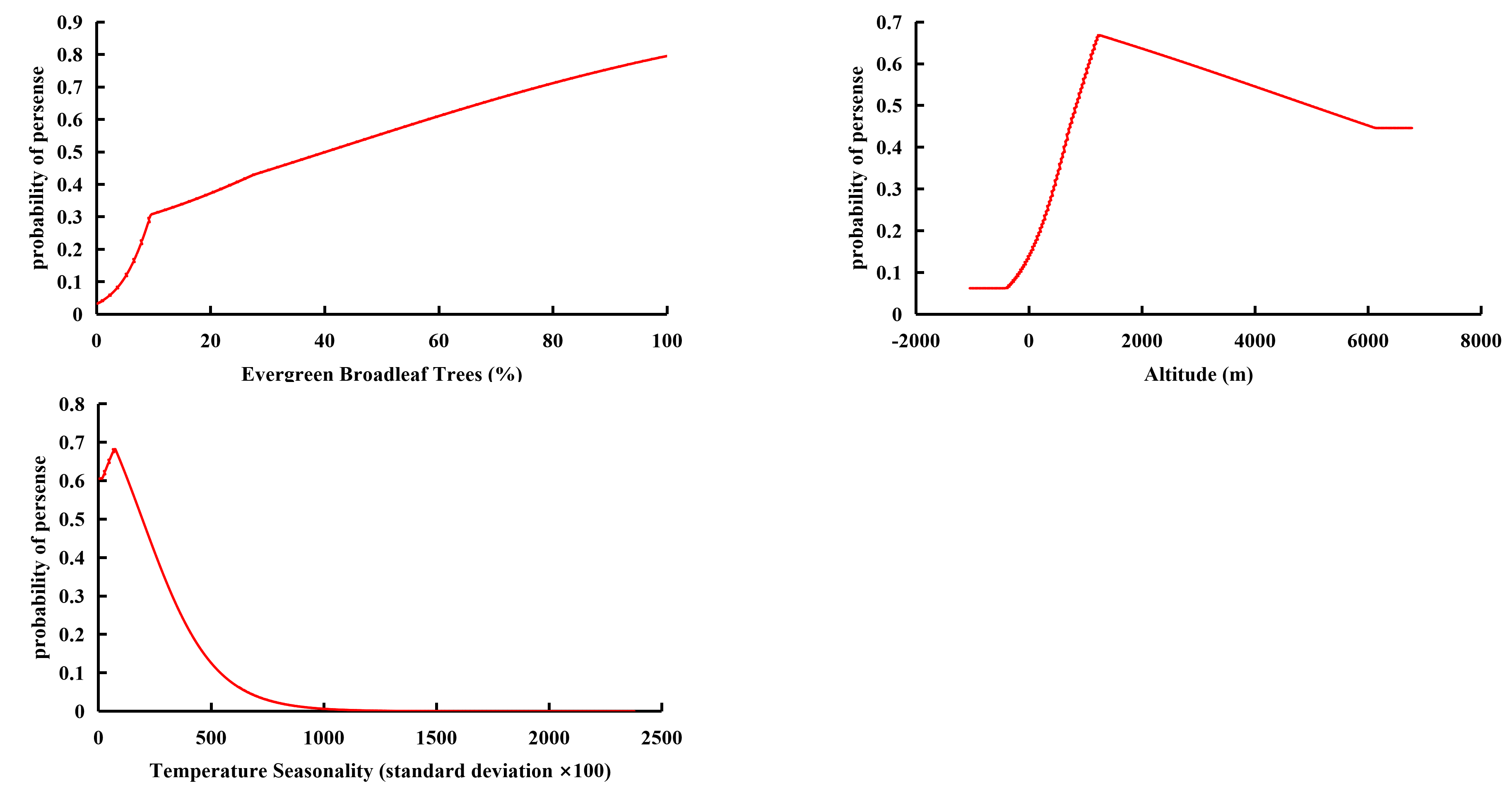


**Figure S8** Response of *P. purpurea* to the four key environmental variables.


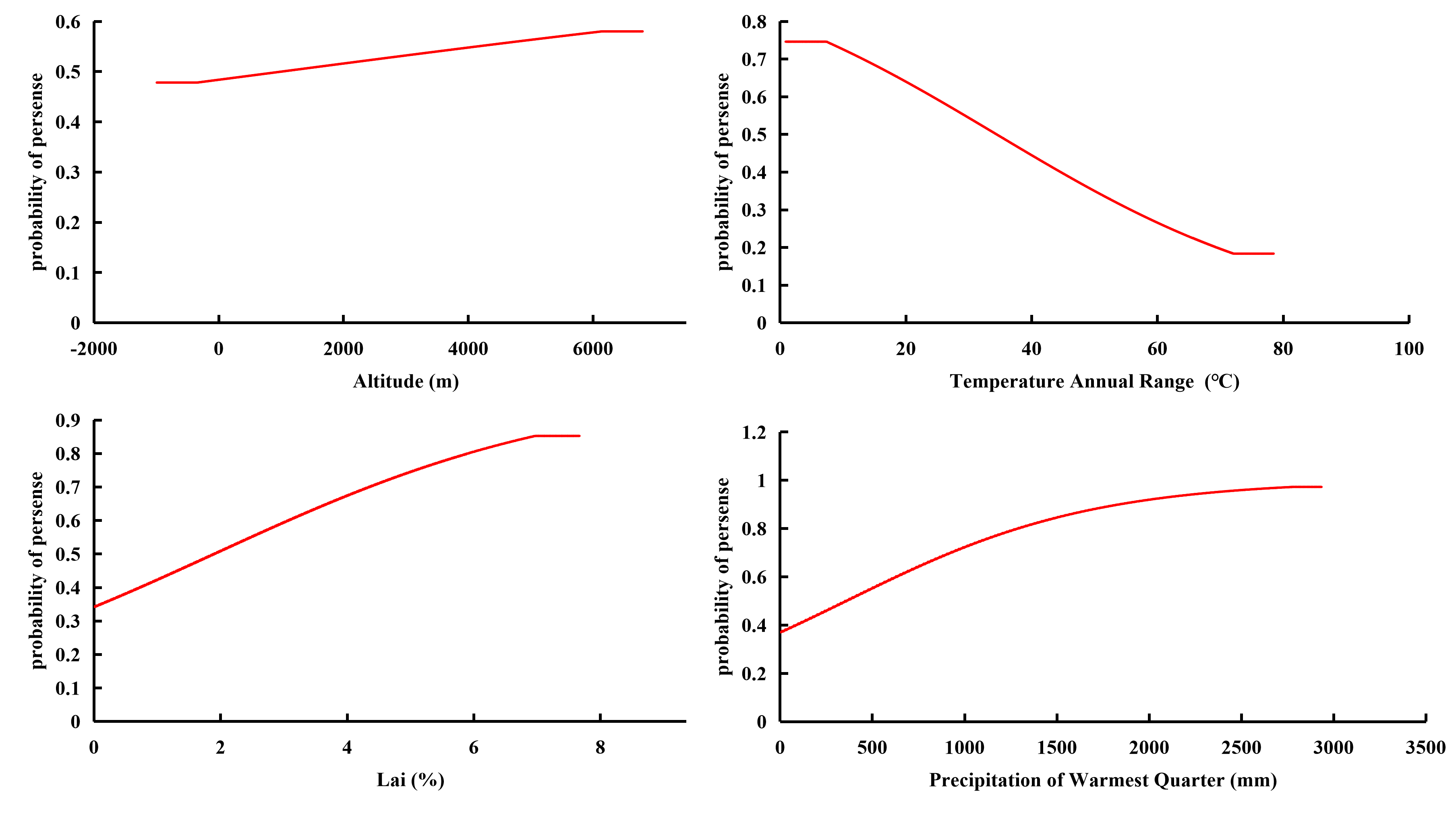


**Figure S9** Response of *P. subinflata* to the three key environmental variables.


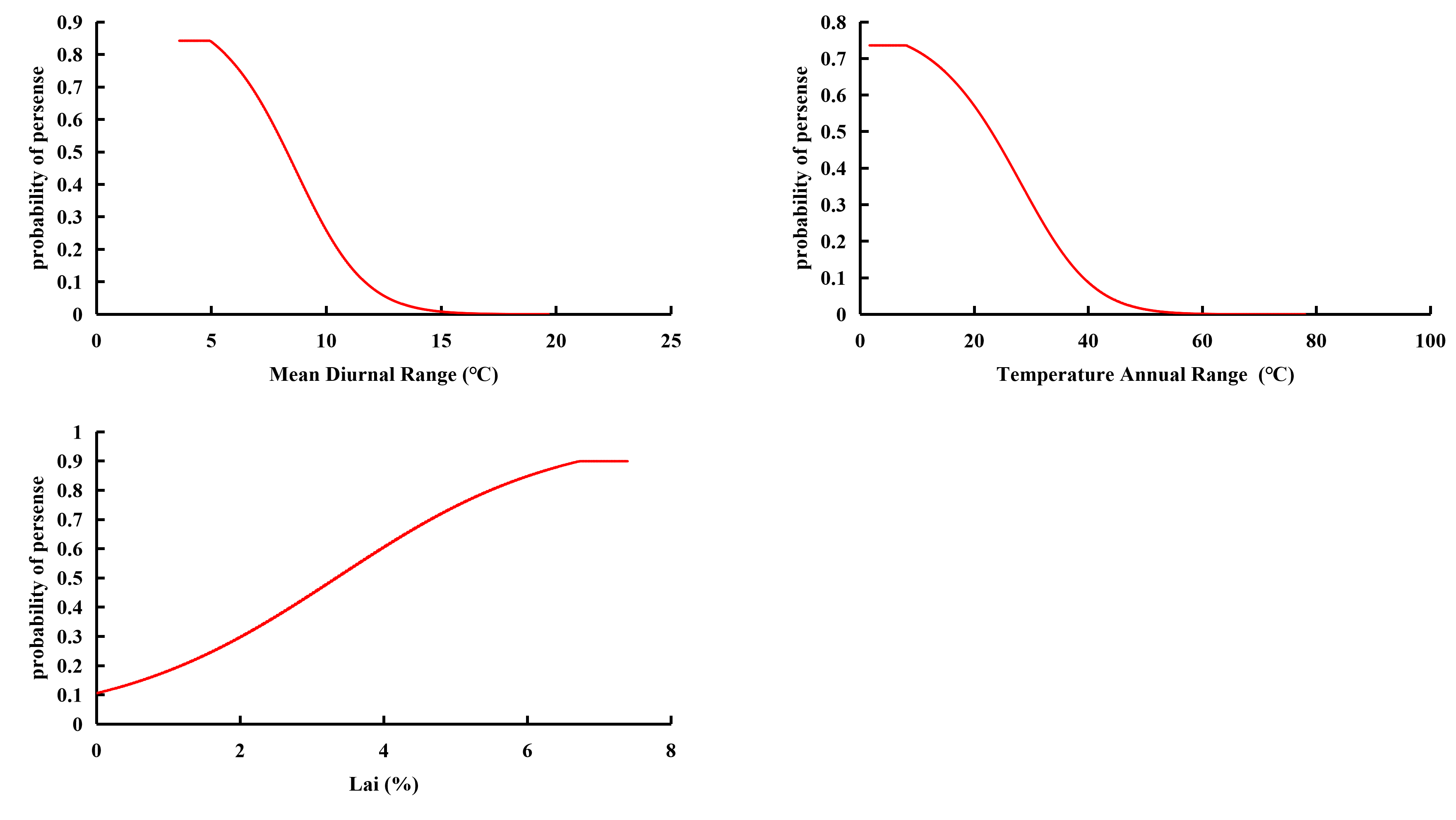

Supplement: Supplementary file 1 — Appendix S1: Details on species occurrence data, environmental variable screening, and MaxEnt modeling results, including Tables S1–S7 and Figures S1–S9. Table S1: 81 valid data points of occurrence used for modeling of this study. Table S2: Screened environmental variables. Table S3: Optimal parameters of MaxEnt model for Pleurozia and four Pleurozia species. Table S4: Dominant environmental variables influencing geographic distribution patterns of Pleurozia. Table S5: Dominant environmental variables influencing geographic distribution patterns of four pleurozia species. Table S6: Changes in the areas (× 104 km2) of suitable habitats for Pleurozia under the current and future climatic conditions. “Total” is the total area of suitable habitats. Table S7: Changes in the areas (× 104 km2) of suitable habitats for four Pleurozia species under the current and future climatic conditions. “Total” is the total area of suitable habitats. Figure S1: Heat map of environmental variable correlations. Figure S2: AUC values of Pleurozia and four Pleurozia species. Note: A: Pleurozia; B: P. subinflata ; C: P. acinosa ; D: P. gigantea ; E: P. purpurea . Figure S3: The Jackknife test results of environmental variables for Pleurozia in Asia. Note: The dark blue bar indicates the gain obtained by using each variable individually, the light blue bar indicates the gain lost by removing a single variable from the whole model, and the red bar indicates the gain obtained by using all variables. Figure S4: Response curves of four environmental factors. Figure S5: The Jackknife test results of environmental variables for four pleurozia species in Asia. Figure S6: Response of P. acinosa to the three key environmental variables. Figure S7: Response of P. gigantea to the three key environmental variables. Figure S8: Response of P. purpurea to the four key environmental variables. Figure S9: Response of P. subinflata to the three key environmental variables. [file ECE3-16-e73657-s001.docx]
